# HRTEM Imaging and Mechanistic Insights Into Carbon Nanotube Nucleation and Growth on Fe Nanocatalysts in a Thermal Plasma

**DOI:** 10.1002/smtd.202502065

**Published:** 2026-04-02

**Authors:** Hengfei Gu, Stanislav Musikhin, Guangming Cheng, Nan Yao, Yevgeny Raitses, Bruce E. Koel

**Affiliations:** ^1^ Department of Chemical & Biological Engineering Princeton University Princeton New Jersey USA; ^2^ Princeton Plasma Physics Laboratory Princeton New Jersey USA; ^3^ Princeton Materials Institute Princeton University Princeton New Jersey USA

**Keywords:** carbon nanotube growth and nucleation mechanisms, face‐centered cubic (FCC) Fe, Fe nanocatalysts, single‐wall or double‐wall carbon nanotube, thermal plasma synthesis

## Abstract

Thermal plasma decomposition of natural gas is a scalable pathway for the production of hydrogen alongside high‐value carbon nanotubes (CNTs). Metals evaporate from an electrode and condense to form seed nanoparticles that nucleate and grow CNTs. However, the lack of mechanistic understanding of the CNT nucleation and growth processes in thermal plasma makes control over CNT diameter, chirality, length, and yield difficult. We debundled and separated CNTs from soots produced using iron (Fe) nanocatalysts, and distributed them on monolayer graphene for high‐resolution transmission electron microscopy (HRTEM) imaging to gain mechanistic insights. Full graphene encapsulation was found for relatively small Fe nanoparticles that were molten at high temperatures. Zigzag single‐wall or double‐wall CNTs (SWCNTs or DWCNTs) appeared to have grown out directly from the graphene covering on the conical or cylindrical bodies of small molten Fe nanodroplets with high curvature. Also, SWCNTs likely grew out from H‐ or O‐atom etched single‐wall carbon nanocones observed on conical Fe nanoparticles. A SWCNT/DWCNT could also be generated from the cracked opening of the graphene covering on a face‐centered cubic (FCC) Fe nanoparticle. A simple, plausible pathway is proposed for the growth of an open, H‐passivated, zigzag SWCNT involving reaction of CH_2_ and CH radicals at high temperatures.

## Graphical abstract

Carbon nanotubes (CNTs) were successfully debundled from Tuball soots produced using iron nanocatalysts in arc discharge thermal plasma and distributed on monolayer graphene for HRTEM imaging for mechanistic insights into CNT synthesis. Zigzag single‐wall or double‐wall CNTs were identified. Multiple models of CNT nucleated on Fe nanocatalysts are proposed and a simple, plausible zigzag CNT growth pathway is proposed.

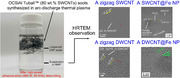

## 1 Introduction

Hydrogen (H_2_) is a good energy carrier for renewable energy sources such as solar and wind [1, 2]. One of the pathways for carbon dioxide‐free production of H_2_ is to use plasma, which can be powered by renewable energy‐based electricity, to decompose hydrocarbons to produce H_2_ and solid carbon. Hydrocarbons can come from natural gas, which is mostly methane (CH_4_) [3]. Techno‐economic analysis indicates that high‐value solid carbon byproducts, including but not limited to graphene flakes, carbon fibers, and carbon nanotubes (CNTs), can offset the cost of using plasma to produce H_2_ [4]. Graphene has high mechanical strength [5], excellent electric conductivity [6], and ultrahigh basal‐plane thermal conductivity [7]. A single‐wall carbon nanotube (SWCNT) can be considered as a structure obtained from curling a monolayer of a graphene sheet into a cylindrical shape and closing the sheet edges via forming C─C bonds. The way in which a SWCNT is rolled into this tubular structure determines its chirality and diameter, giving rise to an electronic structure that can be either metallic or semiconducting. Double‐wall carbon nanotubes (DWCNTs) or multiple‐wall carbon nanotubes (MWCNTs) are made of two or more SWCNTs with different diameters nestled within one another with van der Waals interactions between the cylindrical walls. In 1993, Lijima and Ichihashi first synthesized and reported ultrasmall SWCNTs in a size between 1 and 2 nm using an arc discharge method [8]. Since then, CNTs have been successfully synthesized not only via arc discharge but also through other techniques such as laser ablation [9], plasma torch [10], and chemical vapor deposition (CVD) [11, 12]. Due to the exceptional properties of CNTs, their applications have been intensively investigated for applications in various fields such as electronics, batteries, supercapacitors, catalysis, hydrogen storage, and biomedical applications [13, 14]. While these applications are very important, it seems clear that the only CNT application that will scale to the task of satisfying world demands for low‐cost H_2_ for chemicals and fuels from renewable energy is to utilize CNTs as additives in building or structural materials [15]. But this requires dramatically increasing the energy efficiency and production scale of CNTs.

In research laboratories, a direct current (DC) anodic arc discharge is typically utilized to ablate a graphite electrode in an inert gas such as helium to generate C1 and C2 species to produce CNTs, but can also be used to decompose hydrocarbon feedstocks such as methane (CH_4_) for synthesis of CNTs and the production of H_2_ [8, 16, 17, 18, 19]. In a DC arc, a voltage between 15 and 30 V is applied to two electrodes, where the anode is commonly made of graphite, with a several millimeter interelectrode gap to generate a high current to melt and ablate catalyst materials that were placed within a hole in the anode. The buffer gas can be one of argon, helium, and/or hydrogen. It can also be mixed with a hydrocarbon gas feedstock. The induced quasi‐thermal plasma (when electron, ion and neutral atom and molecules are at or near a thermal equilibrium, that is, the temperature of these plasma species are nearly equal) within the electrode gap can be as hot as 4000–6000 K and decomposes hydrocarbon carbon molecules to form reactive radicals such as CH_3_, CH_2_, CH, and H. CNTs may be formed on the periphery of an arc region at a high temperature (1200–1800 K) [16, 20]. Due to the high‐temperature growth of CNTs in thermal plasma, it is commonly accepted that this method has an appealing advantage for the production of CNTs with fewer structural defects as compared to the other lower temperature synthesis techniques, for example, the CVD method [21].

To facilitate nucleation and growth of CNTs, increase CNT yield, and control CNT diameter, metallic catalysts have been employed in an arc discharge by adding metals on the anode side [8, 22, 23, 24, 25]. These metals are melted by the arc discharge and form metal vapors that condense into nanoparticles (NPs) that serve as nanocatalysts within the plasma and near‐plasma regions. Therefore, these metals must possess relatively low melting and boiling points, have evaporation rates that are neither too high nor too low, and be cost‐effective [17]. To meet these requirements, there are not many choices, and the best candidates so far are iron (Fe), cobalt (Co), and nickel (Ni) [24, 26]. Fe is the most inexpensive and also has a high evaporation rate [18, 19, 27]. Their alloys (e.g., Fe/Co, Co/Ni, Fe/Ni, and Ni/Y) were also investigated for producing CNTs in an arc [24, 25, 28]. For instance, Saito et al. reported that Fe/Ni with a weight ratio of 1:1 was the most effective catalyst for producing SWCNTs among the elemental and binary metals of Fe, Co, and Ni [24], which was consistent with the findings of Seraphin and Zhou [28]. Takizawa et al. found that the mean diameter of produced SWCNTs increased with increasing the concentration of Y within Ni [25]. In addition, Keidar et al. successfully increased the length of SWCNTs (above 5 µm) formed in an arc by using a magnetic field [29]. The thermal conductivity of SWCNTs was experimentally demonstrated to increase with length [30], making the synthesis of long SWCNTs attractive. We note that most of these cited studies were carried out in an inert gas without adding a gaseous hydrocarbon feedstock. Currently, scaling up of the synthesis of CNTs via thermal plasma produced by atmospheric pressure or near atmospheric (so‐called sub‐atmospheric) pressure arc discharge is being conducted by OCSiAl, a company with the world's largest facility for industrial‐scale production of SWCNTs [31, 32, 33]. In this process, it appears from descriptions in their patent and published documents that a hydrocarbon feedstock is used in a sub‐atmospheric DC arc that utilizes liquid‐metal cathodes and anodes that co‐produce Fe nanoparticles that act as catalysts for CNT synthesis.

A goal in our research is to advance our understanding of the mechanisms underlying the nucleation and growth of CNTs on metal nanocatalysts in a thermal plasma generated by a DC arc discharge between two electrodes. We note that a thermal plasma in the form of a jet is not considered in this work. As a useful starting point, we used state‐of‐the‐art high‐resolution transimission electron microscopy (HRTEM) to characterize commercially available OCSiAl Tuball samples of 80 wt.% SWCNT soots to better understand the relationship of the SWCNTs and the Fe nanocatalysts to obtain new mechanistic insights into the synthesis of SWCNTs in this process. Herein, we first describe our use of high‐power ultrasonication directly to debundle the SWCNTs from the soots from Tuball. Fe nanocatalysts with nucleated CNTs were successfully found and imaged at the atomic scale on ultrathin graphene monolayer supports in an HRTEM. Based on our HRTEM observations and molecular dynamics (MD) simulations, we propose mechanisms underlying the nucleation and growth of SWCNTs and DWCNTs on molten Fe nanodroplets or face‐centered cubic (FCC) γ‐Fe (austenite phase) NPs above 1185 K (the temperature for body‐centered cubic (BCC) α‐Fe (ferrite phase) to FCC Fe transformation of pure Fe) [34]. Observations reported herein enable some initial insights into the mechanisms underlying CNT nucleation and growth on metal nanocatalysts in thermal plasma and differences with CNT synthesis using more extensively studied CVD methods. We note that the mechanisms underlying the nucleation and growth of CNTs on α‐Fe or Fe_3_C (cementite phase) at lower temperatures (<1185 K) using the CVD method are not discussed herein.

## 2 Results

### 2.1 Debundling and Characterization of Soots Produced From High‐Temperature Plasma

Carbon soots containing CNTs (Tuball, 80 wt.% SWCNTs), which were presumably synthesized via decomposition of hydrocarbons at high temperatures in thermal plasma produced by arc discharge using Fe as a catalyst were studied herein. A photograph of the as‐received Tuball sample is shown in Figure S1. CNTs concurrently assemble into bundles in the thermal plasma and later due to Van der Waals interactions between the CNT walls, and so it is challenging to debundle CNTs from the soots and find the exact metal NPs that nucleate CNTs among a large amount of NPs formed in the arc discharge for observation at the atomic scale by HRTEM imaging [8]. Debundling of individual SWCNTs from soots was successfully achieved mainly by dissolving metal nanoparticles using acid reflux (HCl, HNO_3_ or HCl/H_2_SO_4_), followed by ultrasonication of the resulting soots in a polar, non‐hydrogen‐boning Lewis bases such as *N*, *N*‐dimethylformamide (DMF) and *N*‐methyl‐2‐pyrrolidone (NMP) and final centrifugation to separate the light and heavy parts [31, 35, 36]. In this study, we used high‐power ultrasonication (480 W, 40 kHz; VEVOR, Model: JPS‐80A) in NMP directly to debundle the as‐received soots containing 80 wt.% SWCNT without using acid to avoid removing the Fe NP catalysts. The ultrasonication time was incrementally increased to 12 h, when we observed isolated CNTs using HRTEM imaging. After ultrasonication, centrifugation was performed to separate the debundled CNTs from the soots.

**FIGURE 1:**
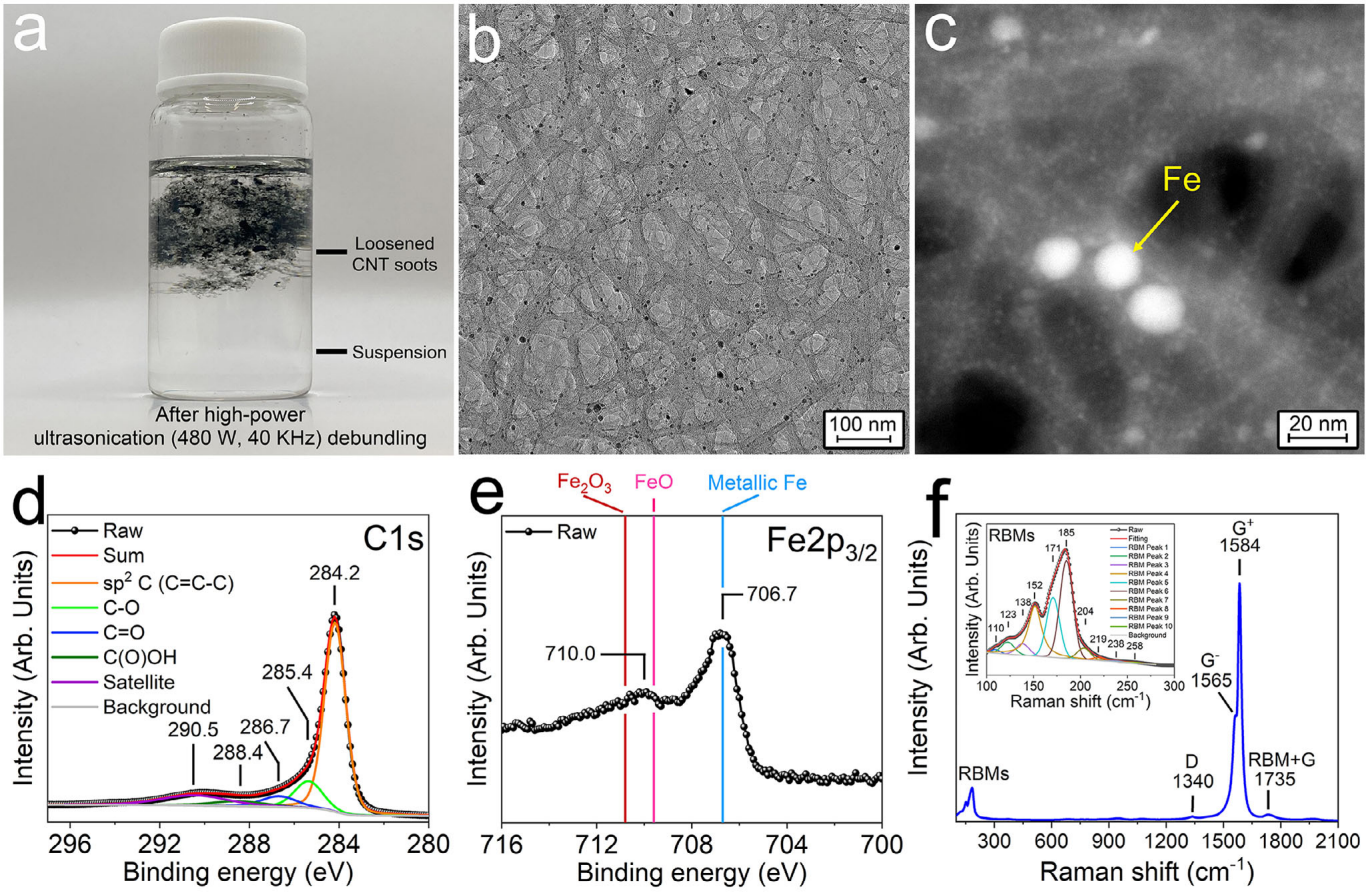
Characterization of soots produced by high‐temperature thermal plasma. (a) Photograph of the soots after high‐power ultrasonication in NMP for debundling and subsequent centrifugation. (b) Bright field TEM image and (c) HAADF image of loosened CNT bundles with Fe NPs after debundling. (d) C1s and (e) Fe2p_3/2_ XPS spectra of as‐received soots on a 15 nm Ni/4 nm TiN/n^+^Si substrate. (f) Raman scattering from as‐received soots on quartz glass. The inset shows a zoomed‐in view of the fitting involved for the low‐frequency CNT RBMs.

Figure 1a shows the loosened soots sitting within the top layer of the NMP solution in a glass vial after ultrasonication plus centrifugation. The thick (middle) and thin (topmost) parts appear as dark and gray colors, respectively. The transparent solution at the bottom of the vial is a suspension composed of separated CNTs, CNT bundles, and Fe NPs, which was revealed by TEM observations described below. After the debundling treatments, the thinned soots were composed of stacked CNT bundles (gray bands) with Fe NPs (dark spots) as seen on a copper grid (without carbon backing) in TEM (Figure 1b). The high‐angle annular dark field (HAADF) image (Figure 1c) demonstrates that the CNT bundles (gray bands) were covered with a large amount of Fe NPs with a size smaller than 5 nm (small bright spots) and some relatively large Fe NPs (larger bright spots). The latter was confirmed by energy‐dispersive X‐ray spectroscopy (EDXS) mapping and point analysis (Figure S2). For X‐ray photoelectron spectroscopy (XPS) analysis, the as‐received soots were placed on 15 nm‐Ni/4nm‐TiN films that were thermally evaporated onto the polished surface of an n^+^Si wafer [37]. All XPS spectra (Figure 1d,e; Figure S3) were referenced to the C1s (284.8 eV) peak for adventitious carbon on the Ni thin film surface. The large C1s peak at 284.2 eV is consistent with the sp^2^ hybridization bonding between carbon atoms in the hexagonal lattice of CNTs [38]. In addition, the Fe2p_3/2_ peak in XPS at 706.7 eV indicates metallic Fe from the Fe NPs, while a small Fe2p_3/2_ peak at 710.0 eV is in the range of binding energies of FeO (709.6 eV) [39] and Fe_2_O_3_ (710.8 eV) [40] was also seen, which can be attributed to Fe_3_O_4_ [41]. Raman spectrum (Figure 1f) of the as‐received soots exhibited split tangential G modes (G^−^ and G^+^) at 1565 and 1584 cm^−1^, respectively, due to strain effects within bent CNT bundles that are associated with the tube curvature and/or electron–phonon coupling. In addition, a small defect‐related D mode at 1340 and radial breathing modes (RBMs) mainly at low frequencies of 152, 171, and 185 cm^−1^ were observed [42, 43, 44]. Hembram et al. and Part et al. assigned RBMs at 153 and 170 cm^−1^ to SWCNTs with diameters of 2.2 and 2.0 nm, respectively, by carrying out density functional theory (DFT) calculations [43, 44]. We note that the Raman spectrum that we measured (Figure 1f) is consistent with the one reported previously for Tuball (80 wt.% SWCNTs) [33]. By using HRTEM imaging, as described below, we confirmed the presence of CNTs with a diameter of 2.2 nm, which should give rise to the observed Raman peak at 152 cm^−1^. The RBM frequency exhibits a linear relation to the inverse of the tube diameter *d* [45] as follows:

(1) $$\omega_{\mathrm{RBM}}=\frac{334\;cm^{-1}\;\mathrm{nm}}{d}\;$$

Using this equation, RBM frequencies of 171 and 185 cm^−1^ arise from CNTs with diameters of 2.0 and 1.8 nm, respectively.

**FIGURE 2:**
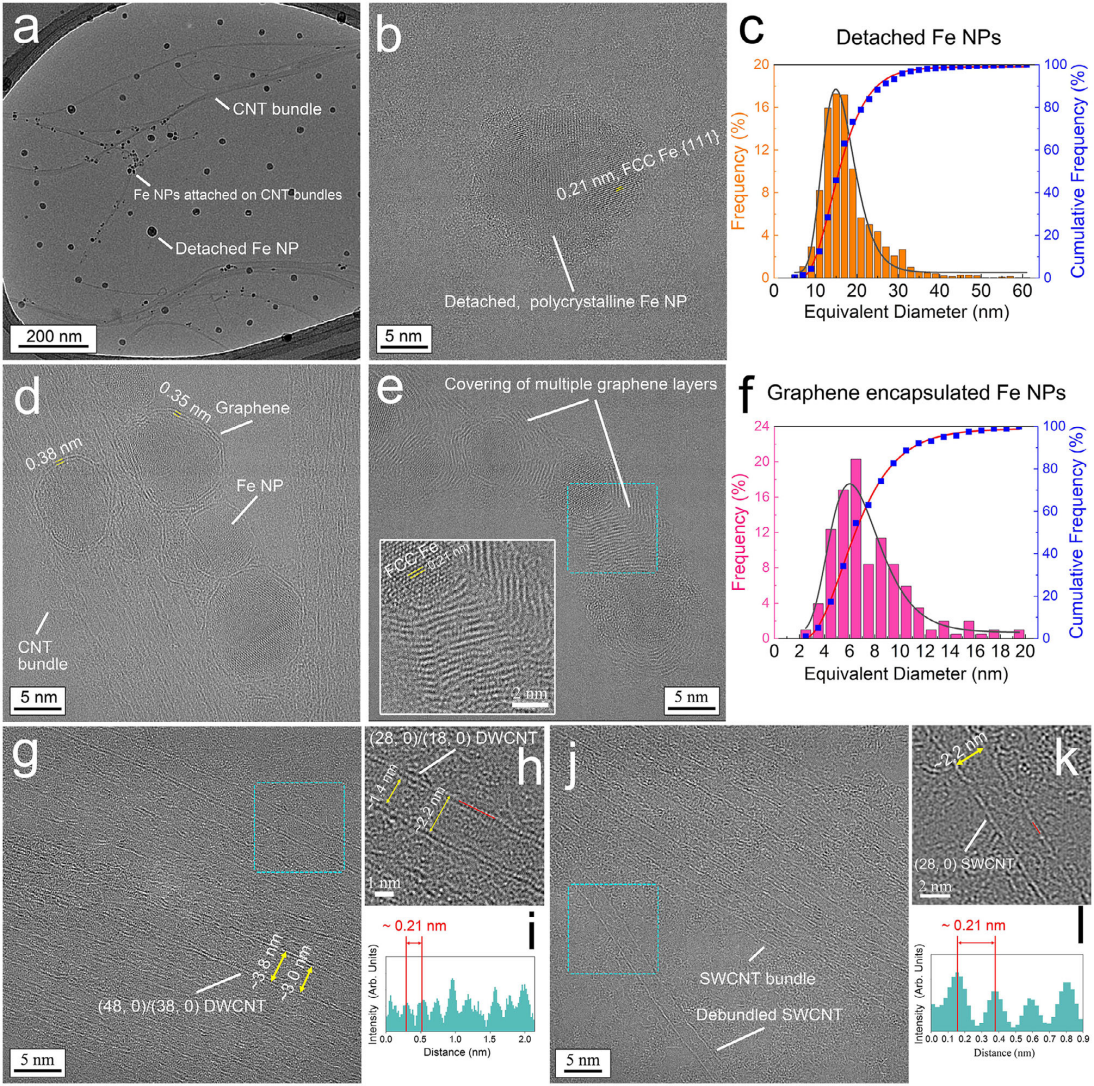
Characterizations of CNTs and Fe NPs that were debundled or detached, respectively, from soots. (a) Bright field TEM image of CNT bundles and Fe NPs after drop casting of the suspension shown in Figure 1a on a monolayer graphene‐backed TEM grid. (b) HRTEM image of a representative, detached Fe NP. (c) Statistical analysis of the size (equivalent diameter) data of detached NPs obtained from the bright field TEM images. Gray and red curves represent the lognormal distribution fit and the lognormal cumulative distribution fit, respectively. (d) HRTEM image of Fe NPs encapsulated by graphene layers that were attached on CNT bundles. (e) HRTEM image of Fe NPs encapsulated by multiple graphene layers. The inset shows a zoomed‐in image of the region boxed in by turquoise dashed lines. (f) Statistical analysis of the size (equivalent diameter) data of the graphene‐encapsulated Fe NPs obtained from HRTEM images. Gray and red curves represent the lognormal distribution fit and the lognormal cumulative distribution fit, respectively. (g) HRTEM image of a CNT bundle containing DWCNTs. (h) Zoomed‐in HRTEM image of the region boxed in by turquoise dashed lines in (g); (i) Brightness analysis of atomic projections on the red line in (h). (j) HRTEM image of a SWCNT bundle and a debundled SWCNT. (k) IFFT image of the region boxed in by turquoise dashed lines in (j). (l) Brightness analysis of atomic projections on the red line in (k).

To investigate the debundled CNTs, as well as the Fe nanocatalysts that nucleated the growth of the CNTs, using HRTEM, the transparent suspension liquid obtained after debundling of the SWCNTs from the soots (Figure 1a) was drop‐casted on a TEM grid coated by a Lacey carbon network that was backed by a monolayer of graphene. HRTEM imaging was performed using a Titan Cubed Themis 300 double spherical aberration (Cs)‐corrected HRTEM at an electron acceleration voltage of 300 kV. To minimize damage due to electron irradiation during imaging [46], HRTEM images were acquired quickly, within a few seconds per image, by moving the electron beam along the CNT bundles. Any images of beam‐damaged CNTs with disordered structures were not included for analysis herein. A low‐magnification bright field TEM image (Figure 2a) shows that the soots were successfully separated into small CNT bundles. This image also identifies relatively large Fe NPs that were detached from the CNT bundles, and smaller Fe NPs that were attached on the CNT bundles. Another HRTEM image (Figure 2b) demonstrates that a representative detached Fe NP was polycrystalline consisting of FCC structures. Also, this Fe NP was likely covered with short C chains and amorphous C. The sizes of the detached Fe NPs from the CTN bundles that were present on bright‐field TEM images were randomly selected and analyzed by manually delineating NP shapes, extracting and coloring them, and measuring their equivalent diameters (denoted as the diameter of a circle with an area equal to that of a NP of interest in a HRTEM image) for statistical analysis (1875 Fe NPs in total, see details in the Supporting Information). The equivalent diameters of the detached Fe NPs (Figure S4a,b) exhibit a lognormal distribution (cumulative frequency: *n* = 29; *χ* ^2 ^= 2.23; *R* ^2 ^= 0.10) with an average of 18.1 ± 0.2 nm (Figure 2c) [47]. HRTEM imaging of the small Fe NPs attached on the CNT bundles reveals that most of these Fe NPs were fully encapsulated by one or two layers of graphene with a spacing between the layers of 0.36 ± 0.1 nm (Figure 2d), which is slightly larger than that between the graphene layers in a graphite lattice (0.34 nm) [48]. Figure 2e shows that in a few cases, we observed graphene layers that had continuously grown thicker and thicker (more than 15 layers) on the hemisurface of a newly formed molten Fe nanodroplet. Similarly, the sizes of the Fe NPs attached to the CTN bundles that were present on HRTEM images were randomly selected and analyzed by manually delineating NP shapes, extracting and coloring them, and measuring their equivalent diameters for statistical analysis (202 Fe NPs in total, see details in the SI). The equivalent diameters of the small graphene‐encapsulated Fe NPs (Figure S4c,d) also roughly follow a lognormal distribution (cumulative frequency: *n* = 18; *χ* ^2^ = 1.86; *R* ^2^ = 0.10) with an average size of 7.6 ± 0.2 nm, which is smaller than that of the detached Fe NPs without full graphene encapsulation (Figure 2f).

HRTEM imaging reveals that DWCNTs were observed (Figure 2g), but SWCNTs made up most of the CNT bundles (e.g., see Figure 2j). Figure 2h clearly shows the atomic structure of a DWCNT with a diameter of 2.2 nm for the outer CNT. The spacing of the atomic projections on the tube edge was 0.21 nm (Figure 2i). These features of the outer CNT are exactly the same as those of a (28, 0) zigzag SWCNT, as Warner et al. revealed using Cs‐corrected HRTEM imaging [49]. To further validate our identification of the CNT chirality, we performed TEM image simulations of a (28, 0) zigzag SWCNT and a (16, 16) armchair SWCNT with a diameter of around 2.2 nm using the QSTEM code (Figure S5) [50]. The very large difference in these simulated CNT TEM images for the same diameters reflects the difference in structures. The match of the simulated (28, 0) zigzag SWCNT TEM image with our obtained TEM images confirms the zigzag chirality of our observed CNTs. In Figure 2j, a SWCNT was separated from a bundle of SWCNTs, and it was also identified as a (28, 0) zigzag type (Figure 2k,l). One can conjecture that during the decomposition of hydrocarbons in a thermal plasma in the presence of Fe nanocatalysts, the growth of (*n*, 0) zigzag CNTs was prevalent. The relationship between the chirality numbers, n and m, and the diameter, d, of a CNT is given in ref [51]. A larger (48, 0)/(38, 0) zigzag DWCNT that was found is shown in Figure 2g. Note that here we did not exclude the possibilities of other types of CNT chirality, which can be studied in the future if they are discovered.

### 2.2 Graphene Encapsulation of Molten Fe Nanodroplets at High Temperatures in a Thermal Plasma

**FIGURE 3:**
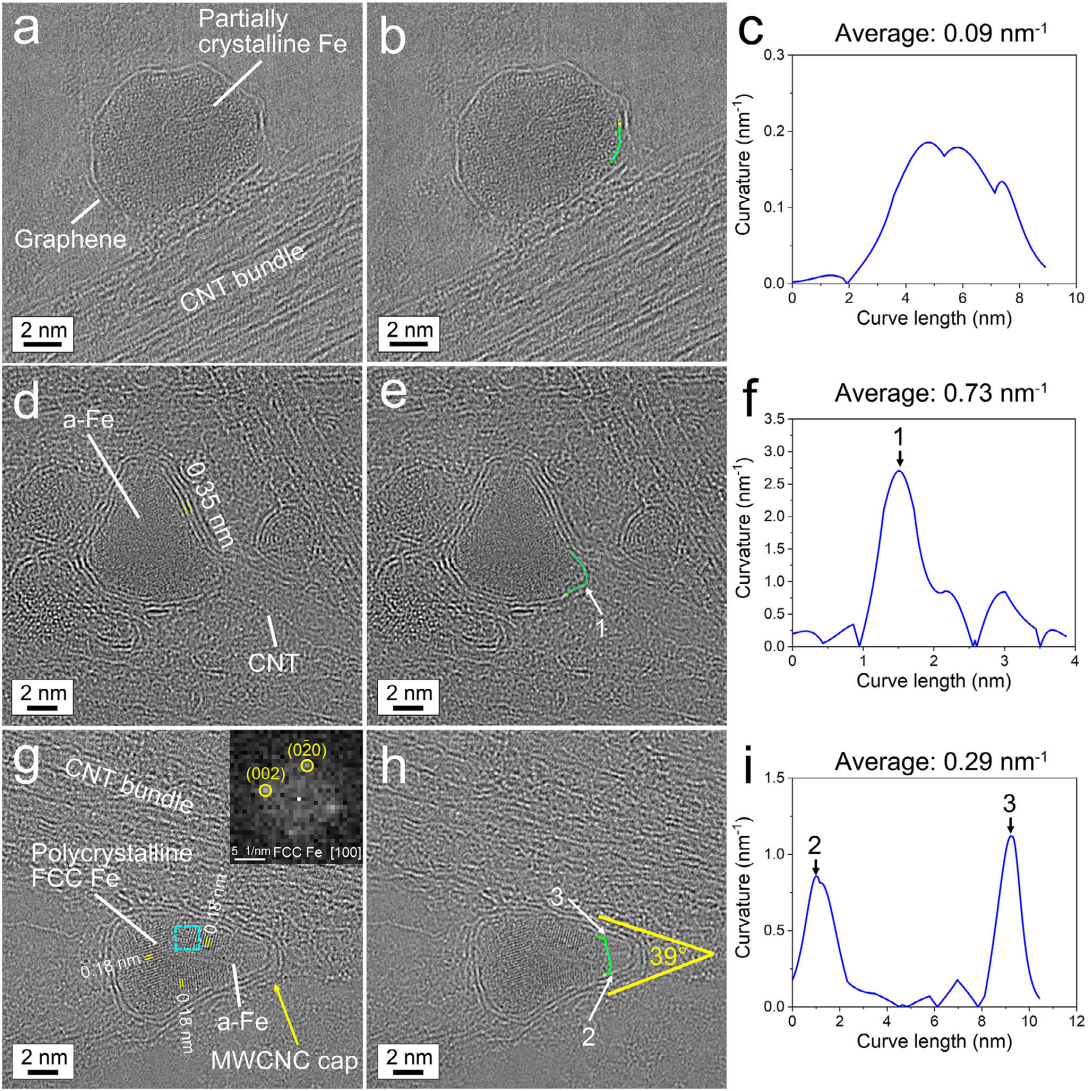
Evidence for graphene encapsulation of molten Fe nanodroplets at high temperatures in a thermal plasma. (a) HRTEM image of a partially crystalline Fe NP encapsulated by a single graphene layer on a CNT bundle. (d) HRTEM image of an amorphous Fe NP encapsulated by multiple graphene layers with a CNT that has grown out at the bottom right corner of the Fe NP. (g) HRTEM image of polycrystalline FCC Fe NP encapsulated by multiple graphene layers with a MWCNC. The inset shows the FFT image obtained from the region boxed in by the turquoise dashed lines. (b,e,h) Same HRTEM images shown in (a,d,g), respectively, but now highlighting the NP surfaces used for curvature measurements composed of green dots, with a single yellow dot as the starting measurement point. (c,f,i) Curvatures from analysis along the green dots highlighted in (b,e,h), respectively.

Figure 3a shows a dew‐like, partially crystalline Fe NP encapsulated by a single graphene layer on a CNT bundle. This Fe NP was flattened at the straight Fe NP/CNT bundle interface, resulting in corners with higher curvatures (Figure 3b,c). Curvature is defined as the inverse of the radius of a circle that is tangent to the surface at a given point. We measured the two‐dimensional (2D) curvatures of Fe NPs captured in the HRTEM images using the open‐source Fiji plugin “Kappa” on the basis of B‐splines [52], and the curvature mentioned below refers to the 2D curvature of the projections of Fe NPs on the HRTEM images unless otherwise noted. Note that 2D curvature is descriptive and not a direct measure of 3D curvature energy or stress. The formation of the straight interface indicates the Fe NP was a liquid nanodroplet when it encountered the CNT bundle. The graphene encapsulation layer is visible at the interface, which suggests that this graphene layer formed on the surface of the molten Fe nanodroplet prior to attachment of the encapsulated Fe nanodroplet on the CNT bundle.

Also, amorphous Fe (denoted as a‐Fe in the figures herein) NPs with graphene encapsulation were found in the HRTEM images. One example is a pear‐like Fe NP encapsulated by three to four layers of graphene in Figure 3d. We suppose that the amorphous Fe structure is formed by quenching of a molten Fe droplet to near room temperature. At the bottom right corner of the NP, a CNT grew out from the graphene encapsulation layers. We note that the NP surface between the two straight CNT walls has a high curvature (an average of 0.73 nm^−1^ and a maximum of 2.70 nm^−1^, as shown in Figure 3e,f), and it is connected to two more straight edges of the NP, where the curvatures are almost zero.

In addition, polycrystalline Fe NPs with graphene encapsulation were observed in HRTEM images. A hazelnut‐shaped Fe NP in Figure 3g comprised three crystal grains with FCC structures that formed from the three relatively straight edges of the NP, and the crystal grain boundaries converged at the center of the NP. The FCC structure of the grains was identified by a fast Fourier transform (FFT) analysis along its [100] zone axis (see inset in Figure 3g). The molten Fe nanodroplet corresponding to this NP presumably underwent annealing in the temperature range of the single‐phase zone of FCC γ‐Fe austenite in the Fe‐C binary phase diagram to crystallize (from 1185 to 1667 K) and then was subsequently quenched to a much lower temperature. As graphene is a good thermal conductor, NP crystallization should occur first at the molten‐Fe nanodroplet/graphene interface followed by the growth of crystals toward the center of the nanodroplet. During crystallization, it appears that a flat surface formed at the right corner of the NP, which was not covered with a graphene layer. On top of this flat surface, a multi‐wall carbon nanocone (CNC) was generated from the graphene layers on the two straight edges of the conical NP body, where the curvatures are approximately zero. We note that at the two junctions connecting the flat NP surface and either of the two flat NP edges (Points #2 and #3 in Figure 3h), the curvatures are very high (maximums of 0.86 and 1.12 nm^−1^, respectively, Figure 3i). It seems that the graphene layers could not cover these sharp junctions with an abrupt increase in curvature leaving the flat, molten Fe NP surface uncovered. Without a graphene covering, it is reasonable to speculate that the heat transfer from the molten Fe to its surrounding environment was poor. This would explain why a small amount of amorphous Fe quenched from molten Fe was found under the flat NP surface. We also note that there is an acute cone angle of 39° between two flat edges of the NP, which determined the shape of the CNC during its formation.

Based on these results, we propose that graphene layers encapsulated the surfaces of molten Fe nanodroplets at high temperatures in the arc discharge. The melting point of graphite incorporating multiple layers of graphene was intensively investigated experimentally at a pressure below 22 MPa and the measured values fell into a wide range between 4000 and 5000 K [53]. Sublimation of carbon atoms from graphite occurs at a much lower temperature, for example, C (gas) pressures of 7.6 × 10^−4^ and 7.6 × 10^−2^ torr can be achieved at 2650 and 3090 K, respectively [54]. Because the melting point of pure iron is much lower (1811 K) [34], the solid graphene/liquid Fe interface may occur at temperatures ≥1811 K. We note that condensed molten Fe nanodroplets with a size below 10 nm would remain in a liquid state at temperatures slightly lower than the bulk melting point [55].

### 2.3 Oxidation of Fe NPs

**FIGURE 4:**
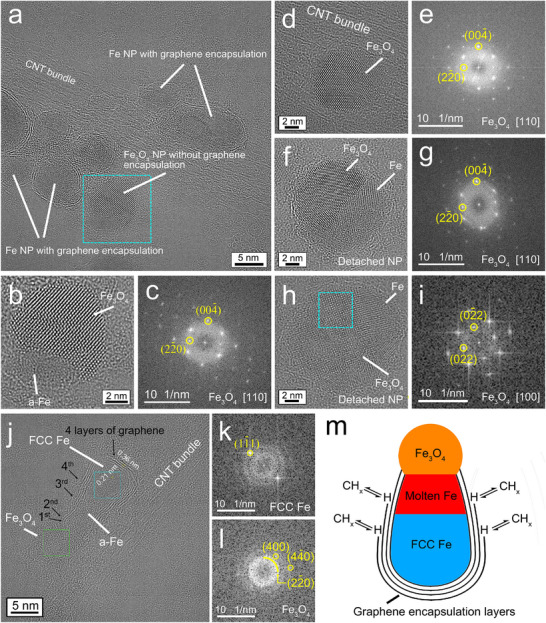
Iron oxide NPs without graphene encapsulation. (a) HRTEM image of a CNT bundle with graphene‐encapsulated Fe NPs and a Fe_3_O_4_ NP without graphene encapsulation. (b) Zoomed‐in image of the Fe_3_O_4_ NP in the region boxed in by the turquois dashed lines in (a). (d) HRTEM image of a different Fe_3_O_4_ NP without graphene encapsulation on a CNT bundle. (f,h) HRTEM images of two separate Fe_3_O_4_ NPs with different orientations detached from the CNT bundles. (c,e,g,i) FFT patterns from analysis of the entire HRTEM images shown in (b,d,f,h), respectively. (j) HRTEM image of a bar‐shaped, partially FCC‐structured Fe NP that had four graphene layers covering its surface and was connected to an amorphous Fe_3_O_4_ NP. (k,l) FFT patterns from analysis of the regions in (j) boxed in by the turquoise and green dashed lines, respectively. (m) Schematic illustration of a proposed bottom–up growth of multiple graphene layers on a Fe NP surface corresponding to the observations in (j).

In addition to graphene‐encapsulated Fe NPs, we observed magnetite iron oxide (Fe_3_O_4_, Fd $\bar{3}$ m space group) NPs attached on or detached from CNT bundles (Figure 4a–i). These were identified by HRTEM imaging followed by FFT analysis along the two zone axes of [110] and [100]. This identification of iron oxides is consistent with that based on the Fe2p_3/2_ XPS binding energy (710.0 eV) in Figure 1e. In Figure 4a, an NP (inside the box of light‐blue dashed lines) next to several graphene‐encapsulated Fe NPs was partially oxidized to Fe_3_O_4_ likely due to the presence of water (H_2_O) molecules in the reactor chamber. Figure 4b shows a zoomed‐in view of this NP. In contrast to the NPs discussed in the previous section, this NP was not covered by graphene layers. Upon additional examination (Figure 4d,f,h), it appears that none of the other Fe_3_O_4_ NPs had a continuous covering of graphene layers. The images in Figure 4b,d,f,h show that the {220} and {004} facets of Fe_3_O_4_ were predominantly terminated by O atoms that have larger atomic projections in the HRTEM images than the Fe atoms, possibly from facile oxidation of surface Fe atoms. This also results in higher O concentrations on the Fe NP surfaces to drive O diffusion into the Fe NPs and oxidation of the bulk Fe NP. Such behavior is in agreement with previous DFT calculations that found that the Fe‐terminated {001} and {111} planes are the most stable surfaces of Fe_3_O_4_, but these terminal Fe atoms will be oxidized under ambient conditions [56]. High concentrations of negatively charged O surface atoms would cause repulsion of the delocalized π electrons of graphene, destabilizing graphene encapsulation on Fe_3_O_4_ surfaces. Without full encapsulation of graphene on Fe NPs, both C and O would more easily diffuse into the outer layer of the relatively large, detached Fe NPs to form Fe_3_C and Fe_3_O_4_, respectively (Figure S6).

### 2.4 Layer‐Layer‐Layer Encapsulation of Molten Fe Nanodroplets and Growth of Open Tubular Graphene Structures

In Figure 4j, an amorphous Fe_3_O_4_ NP (Figure 4l) was attached on one end of a bar‐like, partially FCC‐structured Fe NP that likely resulted from the aggregation and merging of two smaller molten Fe nanodroplets. Two layers of graphene (see first and second labels in Figure 4j) grew on the surface of the Fe NP, but stopped at the contact with the Fe_3_O_4_ NP (and as indicated in the schematic in Figure 4m) due to the unfavorable Fe_3_O_4_/graphene interface. When the graphene layers cannot grow longer, they started to grow thicker, as in the image in Figure 2e. It can be seen in Figure 4j that the third and fourth layers of graphene were shorter (and as indicated in the schematic in Figure 4m); they were growing along the surface of the first two graphene layers from the wider end of the Fe NP toward the Fe_3_O_4_ NP. The Fe NP was partially crystallized in an FCC structure (Figure 4k) beginning at its wider end with thicker graphene layers providing better heat transfer. The two outer cylindrical graphene layers were likely open during their growth in the temperature range for formation of the FCC γ‐Fe austenite single phase of pure Fe (1185–1667 K) or higher [34]. It can be expected that hydrocarbon radicals reacted with and bonded to the carbon atoms at the hexagonal opening of the cylindrical graphene covering (which might be terminated by hydrogen (H)) to extend longer the graphene covering. A corresponding schematic illustration of this proposed process is shown in Figure 4m. These results provide evidence for the free growth of open tubular graphene structures.

### 2.5 Growth of CNCs on Small Molten Fe Nanodroplets and Subsequent Growth of More Stable CNTs From H/O‐Etched CNCs

**FIGURE 5:**
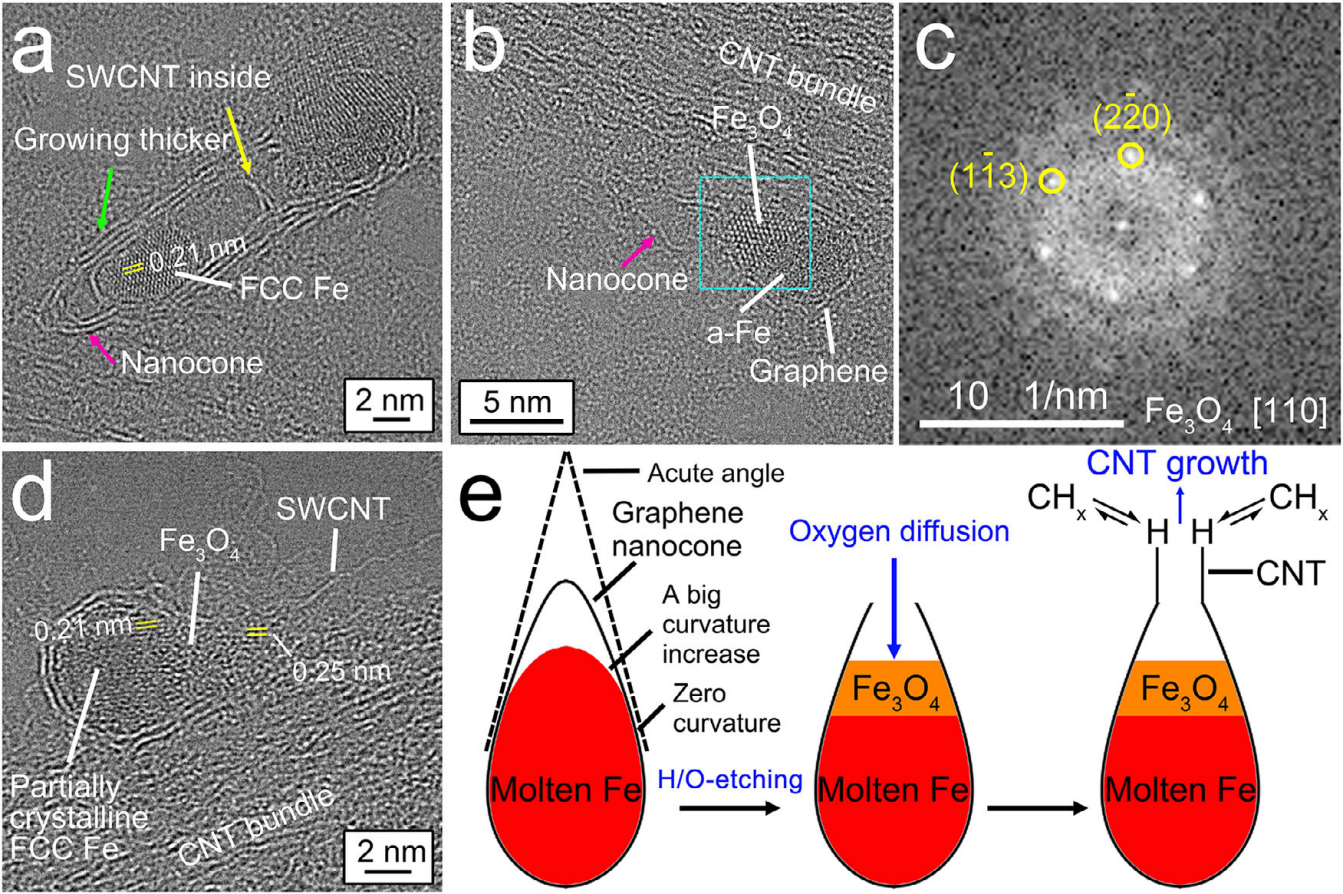
Growth of SWCNTs from an H‐etched CNC grown on small egg‐shaped Fe NPs. (a) HRTEM image of a DWCNC grown on a one‐layer, graphene‐encapsulated FCC Fe NP surrounded by a short SWCNT. (b) SWCNC that grew on an amorphous Fe NP that was partially oxidized. (c) FFT pattern from the region boxed in by the turquoise dashed lines in (b). (d) HRTEM image of a SWCNT that grew on a partially oxidized FCC Fe NP. (e) Schematic illustration of the proposed growth of an SWCNT from an H‐etched CNC on a molten Fe nanodroplet.

Figure 5a shows that an FCC Fe NP, encapsulated by a single graphene layer, with a short SWCNT (at the end of the yellow arrow) on the wider end of the NP, which was encapsulated by additional graphene layers. It can be deduced that the SWCNT growth was stopped after encountering another Fe NP at the upper right corner of Figure 5a and subsequently the NP with a short SWCNT was encapsulated by double graphene layers and ended up with a CNC on the narrower end of the Fe NP (at the end of the red arrow). On the straight graphene surface with zero curvature, one more layer of graphene started to grow (at the end of the green arrow). Thus, it is a logical conclusion that graphene prefers to nucleate on a low curvature surface, particularly on a straight surface (or a cylindrical surface in three dimensions). This FCC NP has a cylindrical body with straight, parallel edges with zero curvature, while the surface of the wider end where the SWCNT grew is relatively flat and has a {100} facet at the very top. At the junctions between the body and the wider end of the NP, the curvatures increase from zero to high values, with a maximum of 2.2 nm^−1^ (see the curvature changes in Figure S7a,b). In this case, a CNT instead of a continuous graphene covering on the NP surface grew out from the two edges of the NP, with the diameter of the CNT equal to the NP size. The narrower end of the FCC NP has smoother corners that enabled encapsulation by the first graphene layer. Formation of the FCC {100} facet bent the graphene layer and led to high‐curvature graphene connections (see the curvature changes in Figure S6c,d). The first graphene layer encapsulation should happen to the molten Fe nanodroplet corresponding to this NP prior to its crystallization. Figure 5a also demonstrates that double graphene layers covered the smoother bottom right corner of the NP. However, covering of the NP by graphene layers stopped where the FCC {100} facet formed with a large increase in curvature (see the curvature changes at Points #4 and #6 in Figure S7c), and a double‐wall CNC (DWCNC) grew out. The largest diameter of the CNC was determined by the width of the FCC {100} termination plane of the NP.

Figure 5b shows an Fe NP of amorphous structure with a guillemot egg (pear) shape that was quenched from a molten Fe nanodroplet with encapsulation by a single graphene layer on its surface and a single‐wall CNC (SWCNC) on its narrower end. The CNC tip appears to be open. The narrower end of the NP where the CNC grew from was oxidized to crystalline Fe_3_O_4_ (Figure 5c). This suggests that the CNC was open and this allowed for oxidant diffusion inside it to oxidize the Fe NP. In contrast, graphene encapsulation can prevent oxidation. The fast growth of graphene structures in an arc discharge has been reported [17], and so it is likely that some CNC growth was completed first. However, because the CNC tip is under high strain and is thermodynamically unstable, it is likely that the tip was subsequently etched away by incident H or O radicals [57, 58, 59, 60]. Figure S8 shows another example of a closed CNC that had grown out on the narrower end of an Fe NP that was fully oxidized to Fe_3_O_4_ from the wider end of the NP that was not covered by graphene. Figure 5d provides an image of another pear‐shaped Fe NP that was partially crystallized to FCC Fe. The wider end of the NP was covered with double graphene layers, while the narrower end of the NP formed a SWCNT. Only the narrower end of the NP, where the SWCNT grew, was oxidized to Fe_3_O_4_. A CNC should form on the narrower end of the NP because of the unparallel edges of the conical NP body and a large increase in curvature when going from the NP body to the ultrasmall end surface. However, no CNC was observed, and so this would be consistent with the tip of the CNC etched away by H or O radicals. At the open end of a CNC, a more stable SWCNT would be expected to nucleate and continuously grow. It would likely take a longer time to oxidize the Fe NP if there were only a low level of oxidants in the reactor or if oxidation was limited by diffusion. Therefore, oxidation of the Fe NP was likely to occur only during the H/O‐etching of the CNC. Once the SWCNT quickly grew out, an oxidant can only diffuse through the inner channel of the CNT, and the longer the CNT is, the longer the oxidant diffusion path is. Oxidation of the Fe NP would be slowed down and stopped during fast CNT growth. A schematic illustration of a proposed process for the growth of a SWCNT via an H/O‐etched CNC is provided in Figure 5e.

### 2.6 Direct Growth of CNTs on Molten Fe Nanodroplets

**FIGURE 6:**
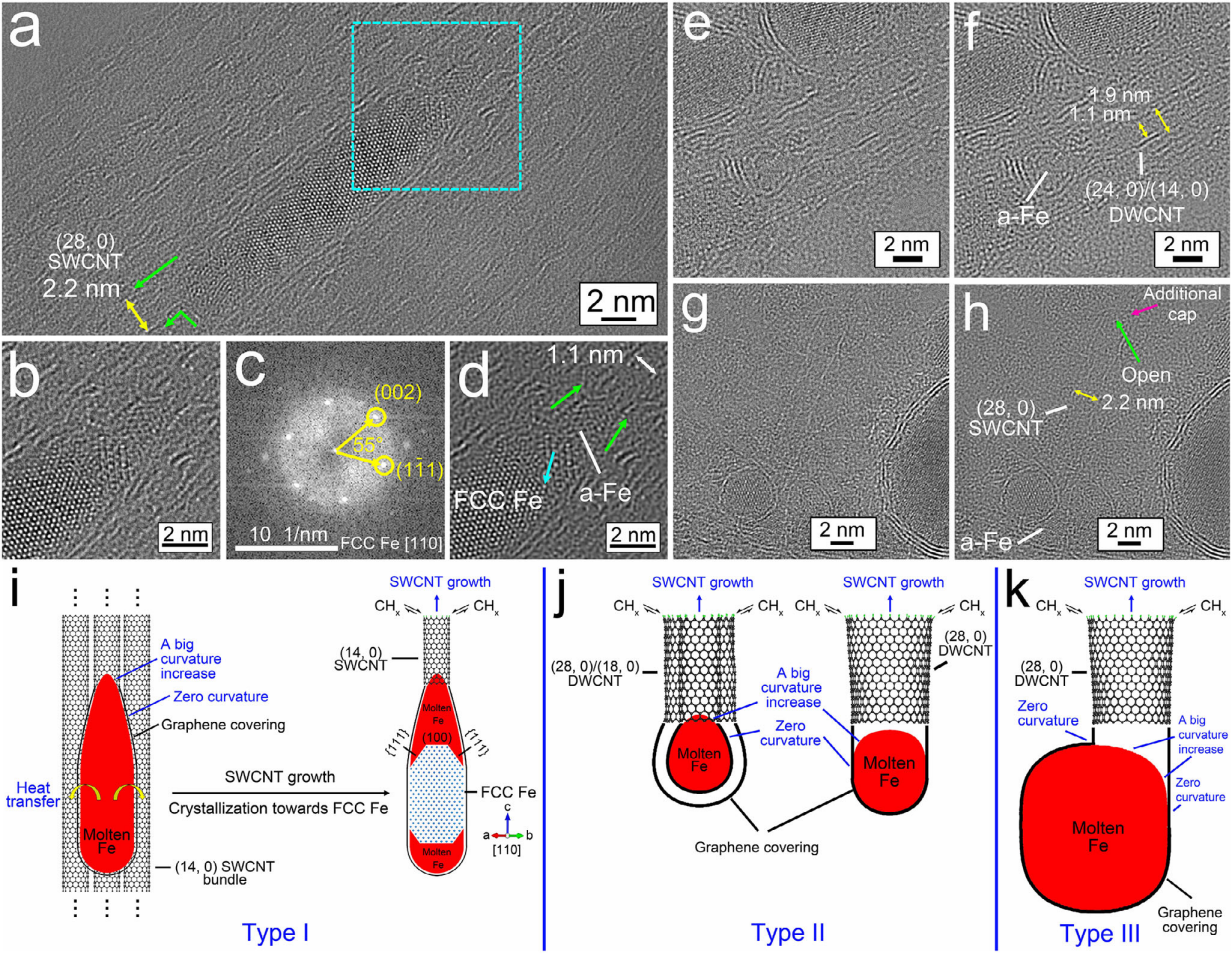
CNTs directly grown on molten Fe nanodroplets. (a) HRTEM image of a partially crystalline FCC Fe NP with SWCNTs that have grown out on its two ends, with the whole structure attached on a CNT bundle. (b) Zoomed‐in image of the region boxed in by the turquoise dashed lines in (a). (c) FFT pattern from analysis of the entire HRTEM image shown in (b). (d) IFFT image corresponding to the image shown in (b). (e) HRTEM image of a DWCNT that was grown on an amorphous Fe NP. (f) IFFT image corresponding to the image in (e). (g) HRTEM image of a SWCNT that was grown on an amorphous NP. (h) IFFT image corresponding to the image in (g). (i–k) Schematic illustrations of the proposed direct growth of CNTs as Type 1 on the sharp conical end of a bullet‐shaped molten Fe NP with its center zone fully crystallized, Type 2 on the ends of ultrasmall conical and cylindrical molten Fe NPs, and Type 3 on one high‐curvature corner of a relatively big, cylindrical molten Fe NP, respectively.

Figure 6a shows a bullet‐shaped Fe NP with SWCNTs grown out of both ends. The central zone of the NP has an FCC structure (Figure 6b,c), while the two ends are partially crystallized with some randomly ordered structures. It can be expected that this solid, elongated NP was formed from a molten Fe nanodroplet undergoing annealing in the temperature range where γ‐Fe is formed, for a short period of time, and subsequent quenching to near room temperature. The FCC single crystal nucleated first in the center of the cylindrical body due to fast heat transfer through the graphene covering at the edges, and continued to crystallize toward the two narrower ends of the Fe nanodroplet. The quenched FCC Fe/liquid Fe interface can be seen in Figure 6b. Figure 6d shows that during the single crystal growth, the most close‐packed {111} facets, which have the lowest surface energy in a FCC structure [61], were in contact with the liquid Fe (light blue arrow in Figure 6d). Such a growth ended up with the {001} planes perpendicular to the parallel edges of the cylindrical NP body, which are parallel to the CNT growth direction. The tip of the sharp conical end has a diameter below 1 nm that has a large increase in curvature (maximum of 0.98 nm^−1^, Point #1 in Figure S9a), and prevents the direct covering of the tip by graphene. This pointed end of the NP was not oxidized. Therefore, the observed SWCNT with a diameter of 1.1 nm, which is assigned to a (14, 0) zigzag SWCNT, should grow directly from the sharp, conical NP end that could be formed by dynamic rearrangements when it was molten (green arrows in Figure 6d). A SWCNT can also grow out directly from the second layer of graphene covering a high‐curvature tip of a sharp conical end of an Fe NP that was already covered by a layer of graphene (green arrows in Figure S10b). This is because the weak Van der Waals attractive force cannot hold two layers of graphene on high‐curvature surfaces. Figure 6i provides a schematic illustration of the direct growth of CNTs on the sharp conical end of a bullet‐like molten Fe NP with its center zone fully crystallized, which is categorized as Type I.

We also found a (24, 0)/(14, 0) zigzag DWCNT grown from the graphene layers encapsulating the narrower end of an ultrasmall egg‐shaped amorphous Fe NP with an equivalent diameter of 2.2 nm (Figure 6e,f). The narrower end of this NP had an average curvature of 0.74 nm^−1^ and a maximum curvature of 1.70 nm^−1^ at its tip (see curvature changes in Figure S9c,d). The smaller the size of a Fe nanodroplet/NP, the larger the curvature of the Fe nanodroplet/NP surface is, making difficult the encapsulation by graphene. Thus, the DWCNT should have grown on this tiny NP when it was molten. Images supporting similar nucleation and growth of a SWCNT on an egg‐shaped molten Fe NP with an equivalent diameter of 3 nm are shown in Figure S11. Figure 6j gives a schematic illustration of the direct growth of CNTs on the ends of ultrasmall conical and cylindrical molten Fe NPs, which is categorized as Type II.

In addition, we observed a short (28, 0) zigzag SWCNT that was 2.2 nm in diameter that appeared to be grown out directly from the single layer of graphene covering a pumpkin‐shaped amorphous Fe NP with almost parallel, straight body edges (Figure 6g). The end of the NP where the SWCNT grew was slightly narrower and had high curvatures on its two corners that connect the two straight NP body edges and the flat surface of the NP end (maximums of 0.56 and 0.58 nm^−1^, Figure S9e,f). These increases in curvature at the NP corners inhibited graphene from covering the NP end surface and led to the nucleation of the SWCNT. The SWCNT itself was likely open (green arrow in Figure 6h), but was capped by an additional flat layer of graphene (red arrow in Figure 6h), which stopped the SWCNT growth. The flat termination will be discussed later in this paper. This growth is also categorized as Type II in Figure 6i.

Additional image details indicate that one more scenario is the growth of an SWCNT at one of the high‐curvature corners of a relatively flat end of an NP. Figure 6a shows a (28, 0) SWCNT that is 2.2 nm in diameter nucleated at a corner of the flat end of a bullet‐shaped Fe NP (green arrows). One wall of the SWCNT is parallel to the graphene covering the straight edge of a cylindrical NP body, while the other wall of the SWCNT is perpendicular to the graphene covering the flat surface of the NP end. The NP corner where the SWCNT grew out has much higher curvatures (maximum of 0.43 nm^−1^) than the straight NP body edge and the flat NP end (Figure S12). In addition, Figure S13 suggests a (18, 0) SWCNT that is 1.4 nm in diameter that also grew on a corner of a pumpkin‐shaped, partially crystalline Fe_3_O_4_ NP, which should be formed by oxidation of a molten Fe NP. One wall of the SWCNT is parallel to the graphene covering the straight edge of the NP body, while the other wall of the SWCNT has an angle of 110° with respective to the graphene covering the flat surface of the NP end. The formation of the oxide indicates the tube was open during its nucleation and growth. The NP corner has a maximum curvature of 0.40 nm^−1^ (Figure S13b,c). In these scenarios, the abrupt, large increase in curvature at the corners of these more cylindrical Fe NPs when they were molten increases the likelihood of nucleating SWCNTs. Figure 6k shows a schematic illustration of the direct growth of CNTs on one high curvature corner of a relatively big, cylindrical molten Fe NP, which is categorized as Type III. Schematic illustrations of all three types of direct CNT growth on the high‐curvature surfaces of Fe NPs when they were molten in the arc discharge are presented together in Figure 6i–k.

### 2.7 Growth of CNTs on FCC Fe NPs After Crystallization of Molten Fe

**FIGURE 7:**
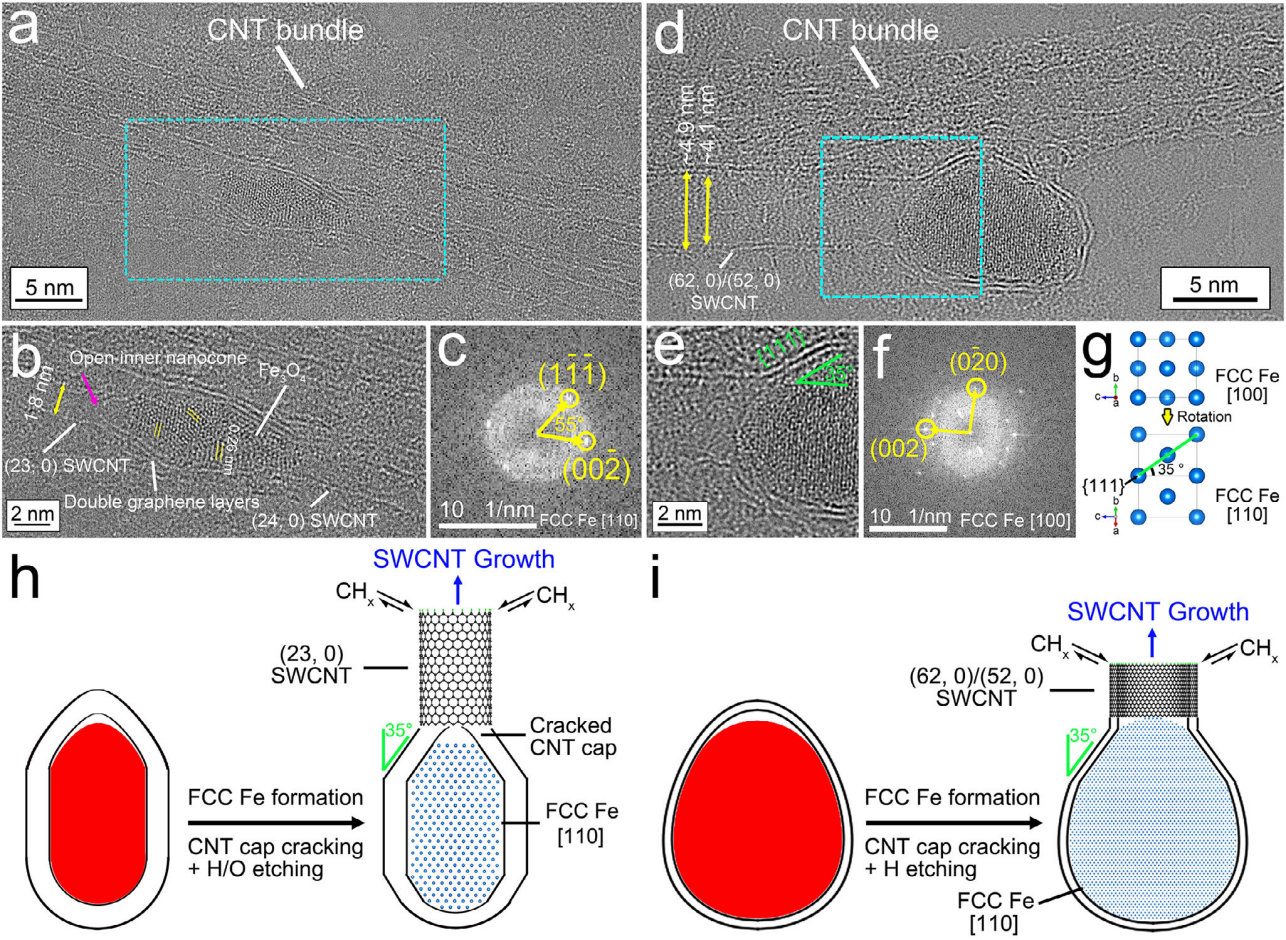
Growth of CNTs on the openings of graphene layers on FCC Fe NPs. (a) HRTEM image of a fish‐shaped crystalline FCC Fe NP with SWCNTs grown out on the two ends with the whole structure attached on a CNT bundle. (b) Zoomed‐in HRTEM image of the region boxed in by the turquoise dashed lines in (a). (c) FFT pattern from analysis of the left‐half part of the FCC Fe NP in (b). (d) HRTEM image of an egg‐shaped crystalline FCC Fe NP that has grown a DWCNT on its narrower end. (e) Zoomed‐in image of the region boxed in by the turquoise dashed lines in (d). (f) FFT pattern from analysis of the entire HRTEM image shown in (e). (g) Schematic of an FCC Fe unit cell viewed along the [100] and [110] zone axes. (h,i) Schematic illustrations of the proposed growth of a SWCNT and a DWCNT, respectively, on the openings of graphene layers on FCC Fe NPs that formed after the graphene layers cracked during crystallization of molten Fe NPs to form FCC Fe.

Figure 7a shows an image of a fish‐shaped Fe NP with SWCNTs that had grown from both ends, which was stuck on a CNT bundle. A zoomed‐in image of this Fe NP is shown in Figure 7b. The SWCNTs on the left and right ends of the NP have diameters of 1.8 and 1.9 nm, respectively, which are assigned to (23, 0) and (24, 0) SWCNTs, respectively. The left part of the NP was fully crystallized in a FCC structure (Figure 7c) with a {001} plane termination at its end and a {111} plane termination on both sides; such terminations lower the overall surface energy. The {001} plane is perpendicular to the SWCNT growth direction, like that for the NP shown in Figure 6a, and the crystallization of the molten Fe nanodroplets within graphene encapsulation should be the same in the two cases. The left part of the NP shown in Figure 7b was covered by double layers of graphene. It can be clearly seen that a SWCNC extended from the inner graphene layer, but was open at its tip (red arrow in Figure 7b). In this image, atomic projections at the open ends of the inner SWCNC are larger than those of the C columns in a crystalline graphene structure, suggesting the presence of clusters of C atoms. This may indicate that the formed inner CNC cracked because of the stresses induced during the preferred growth of {111} planes and the formation of the flat {001} termination by consuming molten Fe, which is like the graphene cracking that can be seen in Figure S14. A (23, 0) SWCNT instead of another CNC grew out from the outer or second layer of graphene. It can be deduced that the (23, 0) SWCNT nucleated on a cracked outer CNC, probably after a small amount of H/O‐etching. Comparing the atomic pattern of FCC Fe within the left part of the NP, the right part possesses larger atomic projections, which suggests the presence of lighter O atoms, and the planes have a larger spacing of 0.26 nm, which is assigned to Fe_3_O_4_ {113} planes. Thus, the (24, 0) SWCNT likely generated from an H‐etched CNC grown on the right end of the NP when it was molten as in the case illustrated in Figure 5d.

Figure 7d shows an egg‐shaped NP with a relatively large equivalent diameter of 8.7 nm that formed a (62, 0)/(52, 0) DWCNT with diameters of 4.9 and 4.1 nm for the outer and inner tubes, respectively, from the double graphene encapsulation layers. The zoomed‐in image of the region where the DWCNT grew from the NP is shown in Figure 7e. This NP was fully crystallized and is identified to be FCC structured. The end of the NP with the DWCNT is terminated with a {001} plane, which is perpendicular to the tube growth direction. On the upper left side of the NP, there is a straight surface with double graphene layers, which extended to one wall of the DWCNT. The normal direction for the {001} planes has an angle of 35° with respect to the straight NP surface. Thus, it can be deduced that the surface is terminated with a {111} facet to minimize the surface energy during crystallization of the molten Fe (Figure 7g). The other wall of the DWCNT was perpendicular to the double graphene layers on the {001} facet. The curvatures of the end of this large NP are not very high (average of 0.31 nm^−1^, Figure S15) and should enable a covering of double layers of graphene. However, the NP end surface between the DWCNT walls was clean, that is, without a graphene covering. One explanation would be that during the preferred growth of the {111} planes in the corresponding molten Fe nanodroplet, the continuous double graphene encapsulation layers on the left end of the Fe nanodroplet cracked, and the DWCNT grew out from the opening of the crack, probably after H/O‐etching of the strained graphene structures after cracking. The proposed corresponding processes for SWCNT and DWCNT growth on FCC Fe NPs in the temperature range between 1185 and 1667 K are illustrated schematically in Figure 7h,i, respectively.

BCC Fe NPs were also observed and identified by FFT analysis along the <111> and <100> zone axes (Figure S16). BCC Fe NPs should be crystallized from molten Fe nanodroplets during annealing in the lower temperature range of α‐Fe ferrite (<1185 K for pure Fe) followed by quenching to near room temperature [34]. The relatively small BCC NPs were fully encapsulated by graphene, but no full graphene covering was seen on the relatively large BCC NPs. During crystallization, {110} facets of BCC Fe formed due to their lower surface energy than {100} facets [61]. When forming a flat {110} facet via consuming molten Fe, a gap might be left under the spherical graphene cover (red arrow in Figure S16a). However, we did not observe BCC Fe NPs that nucleated the growth of CNTs.

### 2.8 Molecular Dynamics (MD) Simulations of the Thermal Stability of CNTs in Vacuum With and Without Hydrogen Passivation

**FIGURE 8:**
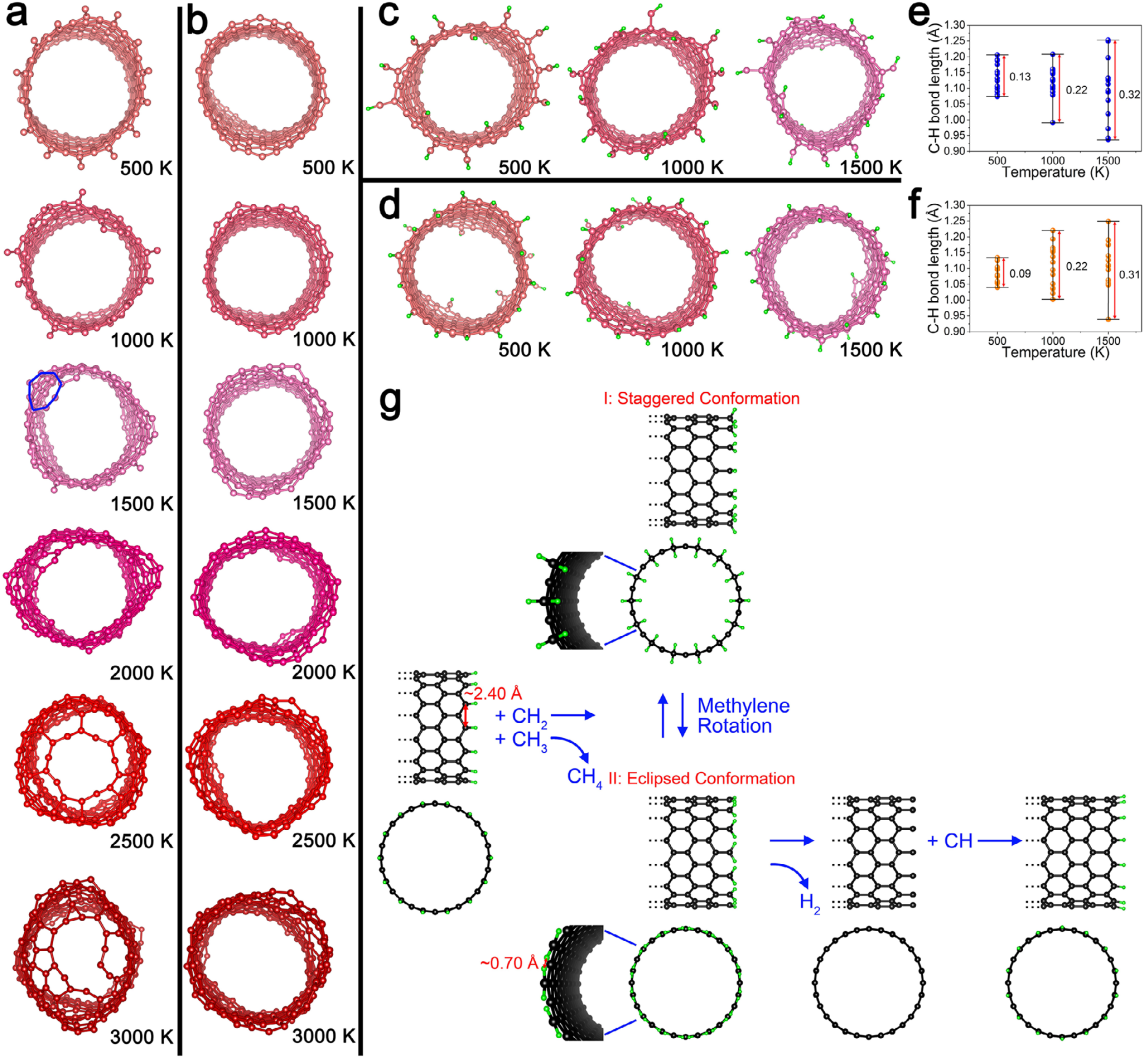
MD simulations of CNTs without and with hydrogen passivation in vacuum and the proposed simplest pathway for growth of zigzag CNTs at high temperatures in the presence of hydrocarbon radicals in a thermal plasma. (a) Perspective views of the C‐zigzag termination of a (14, 0) SWCNT that is 3 nm in length (with the other end in a zigzag termination) after MD simulated annealing in vacuum at temperatures from 500 to 3000 K. (b) Perspective views of the zigzag termination of a (14, 0) SWCNT that is 3 nm in length (with both ends in zigzag terminations) after MD simulated annealing in vacuum at temperatures from 500 to 3000 K. (c,d) Perspective views of the CH‐zigzag termination and the H‐zigzag termination, respectively, of a (14, 0) SWCNT that is 3nm in length (in each case with the same CH‐zigzag termination or a H‐zigzag termination on the other end) after MD simulated annealing in vacuum at temperatures from 500 to 1500 K. (e,f) Instantaneous values of all of the C─H bond lengths at the end of the MD simulation for CH‐zigzag termination and H‐zigzag termination, respectively, as shown in (c,d), for increasing temperatures from 500 to 1500 K. (g) Schematic of the proposed simplest pathway for the growth of zigzag CNTs at temperatures above 1185 K in the presence of hydrocarbon radicals in the thermal plasma.

A (*n*, 0) zigzag CNT has two types of terminations, namely, zigzag terminations with and without dangling carbon atoms. To investigate the intrinsic thermal stability of open zigzag CNTs, we carried out simulated annealing of CNTs 3 nm in length in vacuum within a simulation box 200 Å in *x*, *y*, and *z*‐directions as a function of temperature via MD simulations (1 fs timestep and an NPT ensemble (with constant particle number, pressure, and temperature)) using the LAMMPS code by applying the adaptive intermolecular reactive empirical bond order (AIREBO) potential [62]. The simulated system was equilibrated at 300 K, heated to the target temperature, and maintained for 10 ps. In addition to the reactive empirical bond order (REBO) potential, the AIREBO potential has torsional and van der Waals terms and it has been effectively used to model high‐temperature processes of carbon systems including the melting of graphite and graphene as well as the formation of graphene nanoparticles from carbon chain clusters [63, 64, 65]. Figure 8a,b shows views of a (14, 0) SWCNT 3 nm in length with a carbon (C)‐zigzag termination and a zigzag termination, respectively, after annealing at temperatures ranging from 500 to 3000 K. The corresponding side views of these SWCNTs are shown in Figures S17 and S18. In Figure 8a, for the C‐zigzag termination, the dangling C atoms remain at 500 K. At 1000 K, two adjacent dangling C atoms form a C─C bond that generates a pentagonal ring. At 1500 K, an octagonal ring (highlighted in blue) is observed, suggesting reconstruction of the hexagonal structure with the cleavage and reformation of C–C bonds. At 2500 K, more large rings form and connect to partially cap the tube with a large ring in the tube center. At 3000 K, the large center ring becomes smaller and more pentagonal, and hexagonal rings form to more fully close the cap, suggesting a significant incorporation of carbon atoms from the SWCNT body to the cap. This capping process for the C‐zigzag termination causes a change in the SWCNT heat capacity (the slope of the potential energy (PE) as a function of temperature PE(T) in Figure S19a). In contrast, a SWCNT with a zigzag termination, as shown in Figure 8b, remains open with increasing temperatures from 500 to 3000 K. Furthermore, the open ends of this SWCNT are larger, that is, more extended radially, likely due to thermal expansion. SWCNTs with zigzag termination, without dangling carbon atoms and unpaired electrons, are thermodynamically stable up to 3000 K. In modeling with temperatures up to 6000 K, the heat capacity of a SWCNT with a zigzag termination starts to change significantly at 3015 K (Figure S19b), indicating the beginning of clustering [65]. This temperature is close to the sublimation temperature of graphite [54], which indicates the effectiveness of the AIREBO potential for our MD simulations.

In thermal plasma, both two kinds of SWCNT terminations discussed above could be passivated by H, which would increase their stability by forming CH‐zigzag terminations and H‐zigzag terminations, respectively. Figure 8c,d provides views of the CH‐zigzag termination and the H‐zigzag termination of the same (14, 0) SWCNT at temperatures of 500, 1000, and 1500 K. The corresponding side views of these SWCNTs are shown in Figure S20. At these temperatures, the passivating C─H bonds do not dissociate for either termination. In addition, this hydrogen passivation on the CH‐zigzag termination prevents the formation of large carbon rings at 1500 K that can start to cap the SWCNT. Thus, hydrogen passivation is beneficial for maintaining the open status of the end of an SWCNT. It is important to better understand dehydrogenation at even higher temperatures to consider C─H bonds lengths for the CH‐zigzag terminations and H‐zigzag terminations, as shown in Figure 8e,f, respectively. MD simulations provide information on these C─H bonds lengths, which are shown as instantaneous snapshots of each C─H bond at each temperature. As a reference, the C─H bond length in methane is 1.09 Å. The range of C─H bonds lengths shown in Figure 8e,f shows that the vibrational amplitudes of the C─H bonds increase with increasing temperature. The same MD simulations were conducted for larger (28, 0) SWCNTs (Figure S21) and (24, 0)/(14, 0) DWCNTs (Figure S22) with the more stable H‐zigzag terminations. The same effect of hydrogen passivation on the CNT terminations was found for these additional MD simulations.

## 3 Discussion

We used high‐power ultrasonication of soots in NMP to debundle CNTs. Isolated CNTs were successfully observed (Figure 2j). After ultrasonication, there still existed CNT bundles (Figure 2g,j). This indicates that the ultrasonication was able to break van der Waals interactions, but not powerful enough to enable a full debundling of all of the CNTs, or to break the much stronger covalent bonds within the CNTs. Damage of the structure of individual CNTs due to the applied ultrasonication should be negligible. According to our XPS results, metallic Fe can be maintained over time by the graphene encapsulation or covering, which can prevent oxidation by air. We also observed that many relatively large Fe NPs without full graphene encapsulation after the ultrasonication were not oxidized (Figure 2b). We do not expect that the effect of ex situ oxidation to significantly affect our HRTEM image analysis. As described above, we utilized state‐of‐the‐art HRTEM to analyze structures formed from interactions of CNTs and Fe nanocatalysts in samples obtained from debundled soots from commercially available SWCNTs (Tuball, 80 wt.% SWCNTs), which were presumably synthesized via decomposition of hydrocarbons in a thermal plasma produced by arc discharge using Fe as a catalyst. We note that it is extremely challenging to use spectroscopies such as electron energy loss spectroscopy (EELS) and EDXS to study isolated CNTs and the tiny Fe NPs that nucleated the CNTs because both CNTs and Fe NPs are too thin along the electron beam path in a TEM to provide sufficient signals to produce useful spectra. Based on our HRTEM results, it can be deduced that encapsulation of one, two or multiple layers of graphene happened to relatively small molten Fe nanodroplets (7.6 ± 0.2 nm in average size) at high temperatures (≥1811 K) in the arc, while graphene fragments or amorphous carbon clusters can also form on the surfaces of relatively large molten Fe nanodroplets (18.1 ± 0.2 nm in average size).

Molten Fe nanodroplets can form amorphous, FCC, or BCC structures of Fe after quenching in the reactor chamber. The formation of the resulting room temperature structure depends on the broader temperature gradient in the arc discharge that a Fe nanodroplet underwent prior to the quenching process [66, 67]. Without full graphene encapsulation on the large molten Fe nanodroplets or large crystallized Fe NPs, the surface layers of Fe nanodroplets or NPs might react to form Fe_3_C or Fe_3_O_4_. However, for the Fe NPs we observed, XPS analysis indicated that metallic Fe, rather than Fe_3_C, constituted the dominant chemical nature of Fe. XPS also indicated the presence of Fe_3_O_4_, which is not fully oxidized, and its formation indicates a low H_2_O partial pressure in the reaction chamber. Regarding the lack of forming large amounts of Fe_3_C, we note that Vinogradov et al. successfully grew a single layer of graphene on the (110) surface of a BCC Fe single crystal via thermal decomposition of C_3_H_6_ in a temperature range of 793–873 K at a pressure of 10^−7^ mbar, which is higher than the equilibrium pressure of C atoms for deposition and re‐evaporation (10^−8^ mbar) [68]. The presence of excess carbon feedstock was found to prevent the formation of Fe_3_C and facilitate the growth of graphene sheets, which was confirmed by XPS and low‐energy electron diffraction (LEED). Our observation that little or no Fe_3_C was found within graphene‐encapsulated small Fe NPs is consistent with these results. In both cases, when the carbon feedstock is in sufficient concentration, the coverage of C atoms on the Fe surface is high and increases the likelihood of forming C─C bonds laterally to nucleate and grow a graphene layer. Once a graphene structure is formed, it is very hard to break C─C bonds because of the high C─C bond dissociation energy (590 kJ/mol calculated by DFT) [69] to provide C atoms for diffusion into the Fe subsurface to form Fe_3_C. Thus, a stable graphene covering can block extensive carbidization and oxidation of Fe NPs in the reactor. A graphene covering can also prevent or slow the oxidation of the Fe NPs in our samples at room temperature although they were exposed to ambient air during transfer and storage.

Thermodynamically, condensation of Fe vapor should prefer to form spherical molten nanodroplets at high temperatures in a thermal plasma to reduce surface energy, which is indicated by the observed round shapes of most solidified Fe NPs, especially the relatively large Fe NPs. However, the fine details of the shapes of molten Fe nanodroplets can be changed due to several factors such as complex convection flow [70], particle collision and aggregation, wetting of CNTs, crystallization of molten Fe, and reshaping of the molten Fe NP during CNT growth. Consequently, our observations showed several forms of elongated Fe nanodroplets that had either a cylindrical body with parallel straight edges or a conical body with non‐parallel straight edges forming an acute cone angle, or sharp NP ends with high curvatures. Most of the round, small NPs were entirely covered with one or two layers of graphene and did not appear to grow CNTs. The interfacial energy between two stacked graphene layers was experimentally measured to be in the range of 30–200 mJ/m^2^ [71, 72, 73], which is consistent with later DFT calculations and MD simulations [74, 75]. This weak van der Waals force interaction apparently lowered the likelihood of continuous layer‐by‐layer growth of graphene on small round NPs with relatively high curvatures. Our images showed that all CNCs and CNTs appeared to have grown out from the graphene surrounding the reshaped Fe NPs, suggesting that the presence of graphene on these molten Fe nanodroplets is needed for the nucleation of CNCs or CNTs. Because we observed many small, spherical Fe NPs that were fully covered by graphene layers, and second, we did not observe CNTs attached to Fe_3_C NPs, we propose that these Fe NP surfaces do not play a role in which they act as a sink for C atoms from hydrocarbon decomposition and act as a source feeding C atoms to grow CNCs or CNTs, which is commonly proposed as the root‐growth model in the CVD synthesis of CNTs [21]. If so, then hydrocarbon radicals must impinge and react at the openings of the graphene encapsulation of the NP to grow CNCs or CNTs. In additional images, we found both CNCs and CNTs appeared to have nucleated on localized surfaces of molten Fe nanodroplets where there existed a large curvature increase. This large curvature increase arises from ultrasmall NP ends or rounded corners. To continuously cover an NP surface that has a significant increase in curvature requires changing the growth direction of hexagonal graphene structures by forming strained pentagonal carbon structures like the capping process shown in our MD simulations (Figure 8a; Figure S17). Instead, “sprouting” or local formation of CNCs or CNTs is more energetically favored. The curvature of the circular cross sections of a CNC increases significantly when reducing the radius of the cross‐sectional rings toward the CNC tip pentagon. This high curvature causes large internal strain energies induced by Van der Waals repulsions. The smaller the cone angle of a CNC is, the lower the thermal stability of the CNC. This is why CNCs are somewhat less favorable thermodynamically than CNTs. MD simulations by Tsai and Fang demonstrated that C atoms within CNCs can move out of their original positions at ∼1700 K, leading to defects, and at higher temperatures, the closed CNC cap breaks down and forms a tubular cluster [76]. Therefore, at the high temperatures in the thermal plasma, it is reasonable that the CNCs are able to quickly grow on the conical ends of molten Fe nanodroplets, and then have a good possibility that their caps break down and/or be etched by H or oxidant radicals to allow for nucleation and growth of more stable CNTs. It has been experimentally demonstrated that oxidation of a CNC would start from the pentagons of high strain within the hexagonal graphene lattice of the CNC [60]. On the other hand, if the molten Fe nanodroplet had ultrasmall ends or corners with high curvatures, it is more likely that the CNTs extended and grew out directly from the graphene layers surrounding the conical or cylindrical bodies of these Fe nanodroplets. Note that large Fe NPs without full graphene encapsulation may potentially serve as a source to grow CNTs on top of their surfaces according to a root‐growth model. However, in the Tuball sample, we did not observe any CNTs that were attached to large or low‐curvature Fe NPs without full graphene encapsulation.

As the straight body edges of an elongated conical or cylindrical molten Fe nanodroplet have zero curvatures, these edges would be covered by graphene first, minimizing the internal strain energy within the graphene layer. Due to the exceptional basal‐plane thermal conductivity of graphene, heat would transfer from the body center to the two ends of a molten Fe nanodroplet and result in the crystallization of molten Fe first in the body center of the Fe nanodroplet. The solid/liquid interfaces within the molten Fe nanodroplet would subsequently move toward the two ends. At these interfaces, multiple {111} facets, which have the lowest surface energy for FCC crystals, formed. When the crystallization is completed, a flat {100} facet could be left on one end of the resulting, solidified Fe NP. This result was observed for multiple FCC Fe NPs imaged in our studies. The preferred growth of {111} facets along specific directions in the Fe NP would cause cracking of the graphene covering and create the abrupt interfaces with high curvatures between {111} and {110} facets. Consequently, more stable CNTs would nucleate where the curvatures are high on the FCC Fe NP surface from the opening of the cracked graphene layers.

Zhong and Hong performed MD simulations of the formation at 3500 K of graphene clusters, graphene sheets, and graphene balls from H and hydrocarbon radicals, including CH_3_, CH_2_, and CH [77]. Their study revealed that carbon chains were generated first, and then cyclization of the carbon chains occurred. C atoms at the ends and branches of the carbon chains were always connected to H atoms due to the formation of strong C─H bonds. Extension or cyclization of the carbon chains proceeded with the substitution of C─H bonds for C─C bonds. Hexagonal graphene structures formed and extended along the carbon chains or rings, creating graphene sheets and even graphene balls after curling and closing of the graphene sheets. The addition of excess H radicals prevented the closing of graphene sheets, which could be due to an increased hydrogenation of hydrocarbon radicals with an increasing concentration of H radicals, making the formation of C─C bonds and especially their strained counterparts more difficult. Experimentally, Wang et al. observed open ends of MWCNTs that were produced in a hydrogen arc [57]. Therefore, hydrogen passivation and hydrogen etching by H radicals should prevent closing or capping of CNTs during their growth.

Our simple, qualitative MD simulations revealed that the amplitudes of C─H bond vibrations increase with increasing temperature for both the CH‐zigzag termination and H‐zigzag termination of CNTs (Figure 8e,f), which should facilitate dehydrogenation, namely, breaking the C─H bonds on the terminations to regenerate hydrocarbon molecules or radicals and subsequent formation of C─C bonds. In Figure 8g, we illustrate schematically our proposed simplest pathway for the growth of (*n*, 0) zigzag CNTs using a (14, 0) SWCNT as an example. We start with the H‐zigzag termination of a CNT as it is more stable than a C‐zigzag termination. To extend the length of the CNT, C─H bonds would be substituted by C─CH_2_ bonds with strong sp^2^ hybridization, accompanied by regeneration of CH_4_ (Figure 8g). On the zigzag termination, two adjacent C atoms have a relatively large distance, for example, 2.04 Å for the (14, 0) tube, exposing a large enough space to accept the addition of a methylene (CH_2_) radical. This is likely the reason why the growth of the zigzag type of CNTs was prevalent in the thermal plasma. At high temperatures, the methylene groups keep rotating on the zigzag termination to form the two most stable conformations, namely staggered and eclipsed conformations between the methylene groups and the CNT. To form the eclipsed conformation, two H atoms from two adjacent methylene groups would approach close enough to form H_2_ molecules with an H─H bond length of 0.74 Å. This dehydrogenation results in dangling C atoms. With the addition of CH radicals, the stable CH‐zigzag termination is expected to form again and extend by one more layer the hexagonal graphene structure of the CNT. In our proposed reaction pathway, CH_2_ and CH radicals are the main feedstocks, which is consistent with the MD simulation results of Zhong and Hong that CH_2_ and CH were consumed fast and simultaneously when graphene structures started to generate from hydrocarbon radicals [77]. The more facile dissociation of C─H bonds on the zigzag terminations at high temperatures and the self‐dehydrogenation to form H_2_ via rotation of the methylene groups can help to explain the fast growth of CNTs in thermal plasma. The calculation of the binding energies of CH_2_ radicals on the zigzag CNT termination using DFT would be of interest, but such a calculation is very challenging, in part because radicals are open‐shell species with unpaired electrons. Importantly, at the high temperatures (1200–1800 K) in a thermal plasma, the activation barriers for the substitution of C─H bonds with C─CH_2_ can be overcome. In the future, quantitative MD simulations of the corresponding kinetics with the presence of H_2_/CH_x_ radicals may be performed using a modified AIREBO force field or a reactive force field (ReaxFF) force field at ns timescales.

One‐layer and double‐layer graphene encapsulations were mostly observed on the quenched small Fe NPs, and this can be ascribed to the strong graphene‐Fe chemisorption, weak Van der Waals interactions between graphene layers, and the high curvatures of small Fe nanodroplets. SWCNTs can grow out either from the first layer of graphene or from the second layer of graphene, while DWCNTs necessarily grow out from two layers of graphene. This may explain the high selectivity in the yield of SWCNTs in the thermal plasma produced by an arc discharge using Fe nanocatalysts. We note that CNT growth would be stopped by closing the end of the tube. However, a relatively flat graphene layer rather than fullerene‐like or conical cap was found to form at the ends of CNTs grown out from the cylindrical bodies of Fe NPs imaged in our study (Figures 5a, 6g; Figures S10b and S23). We can explain that by assuming that when the CNT could not grow longer, an additional graphene layer started to grow on the flat termination and eventually developed thicker layers (Figure S10b). It was previously reported that relatively flat, graphene‐layer terminations were observed on MWCNTs synthesized in an arc, as well as short, small SWCNTs and SWCNCs that grew out from the outermost graphene layer covering the high‐curvature end of big MWCNTs synthesized in an arc [60, 78]. In these cases, pentagons existed in the cylindrical CNT‐wall ridges. It is likely that the flat graphene‐layer termination with zero curvature has less internal strain energy than the caps that have high circular curvatures. We note that the presence of pentagons on the cylindrical CNT‐wall ridges induces a small amount of internal strain. Thus, the flat termination of a CNT is favored in thermal plasma if a sufficient supply of hydrocarbon radicals cannot be sustained. Once a CNT is closed, it cannot grow longer.

## 4 Conclusions

By using HRTEM imaging, we characterized commercially available OCSiAl Tuball samples of 80 wt.% SWCNTs. These materials were presumably synthesized using thermal plasma produced by arc discharge to decompose hydrocarbons in the presence of Fe catalysts, which is indirectly supported by our HRTEM analysis. We have developed a better understanding of the relationship of the SWCNTs and the Fe nanocatalysts and have obtained new mechanistic insights into the synthesis of SWCNTs at high temperatures in a thermal plasma. We have revealed herein that in an arc, Fe vapor condenses in the discharge to form mainly spherical Fe nanodroplets to minimize the surface energy. Many of the smaller molten Fe nanodroplets become fully encapsulated by one, two, or multiple graphene layers formed quickly by consuming hydrocarbon radicals. Complete graphene encapsulation blocks or inhibits diffusion of C and oxidant species into the Fe nanodroplets to form Fe_3_O_4_ and Fe_3_C, respectively. Because of the complex convective flows in an arc, along with a variety of other processes that can produce structures other than round droplets, some of the molten Fe nanodroplets generated conical and cylindrical bodies. Graphene, covering the conical body of an ultrasmall molten Fe nanodroplet with high curvature on its end, could extend and generate a SWCNC quickly at high temperatures (≥1811 K). The presence of localized H and/or oxidant radicals may etch the SWCNC, leading to the growth of a more stable SWCNT from the open CNC. In addition, a SWCNT or DWCNT could grow out directly from the conical or cylindrical body of a molten Fe nanodroplet where there exists a big increase in curvature at the nanodroplet end, or a SWCNT could grow out from a sharp corner of a molten Fe nanodroplet with high curvatures. Where there is a large increase in curvature of a Fe nanodroplet surface, the continuous covering by graphene is interrupted and results in the nucleation and growth of CNTs. Depending on the temperature in the broad temperature gradient of an arc, molten Fe nanodroplets were able to crystallize into FCC or BCC structures in solid Fe NPs. Due to the preferred growth of {111} planes during crystallization of molten Fe to FCC Fe at temperatures between 1185 and 1667 K, the graphene encapsulating the Fe NP might crack, and a SWCNT or DWCNT could grow out from this opening. The SWCNTs and DWCNTs produced in the high‐temperature thermal plasma have a zigzag chirality. Our MD simulations indicate that hydrogen passivation on the zigzag termination at the end of a CNT prevents the closing of the end of the CNT. To plausibly explain our HRTEM images of CNTs associated with Fe nanocatalysts, we propose a simplest pathway for the growth of a zigzag CNT that includes steps of substitution of C─H bonds with C─CH_2_ bonds, rotation of neighboring CH_2_ groups for dehydrogenation, and the addition of CH groups. We also suggest that increasing the formation of ultrasmall molten Fe nanodroplets with a conical or cylindrical body in an arc may be important for nucleation and growth of SWCNTs. Observations reported herein indicate mechanistic differences from those for the more extensively studied CVD methods at lower temperatures (<1185 K). The insights gained into the mechanisms underlying nucleation and growth of CNTs on Fe nanocatalysts in thermal plasma will help to guide and improve the production of CNTs at scale in thermal plasma produced by arc discharges.

## Author Contributions

Hengfei Gu: conceptualization, methodology, formal analysis, validation, investigation, data curation, writing the original draft, and writing the review & editing. Bruce E. Koel: conceptualization, methodology, formal analysis, writing the review & editing, supervision, project administration, and funding acquisition. Yevgeny Raitses: conceptualization, formal analysis, writing – review & editing, project administration, and funding acquisition. The other authors: formal analysis, investigation, data curation, and writing the review & editing.

## Conflicts of Interest

The authors declare no conflicts of interest.

## Supporting information




**Supporting File**: smtd70622‐sup‐0001‐SuppMat.pdf.

## Data Availability

The data that support the findings of this study are available from the corresponding author upon reasonable request.
