# Supplementary material for: HRTEM Imaging and Mechanistic Insights Into Carbon Nanotube Nucleation and Growth on Fe Nanocatalysts in a Thermal Plasma
Source: Small Methods. 2026 Apr 2;10(9):e02065. doi: 10.1002/smtd.202502065 (PMC13159396; doi:10.1002/smtd.202502065)
Supplement: Supplementary file 1 — Supporting File: smtd70622‐sup‐0001‐SuppMat.pdf. [file SMTD-10-e02065-s001.pdf]

HRTEM imaging and Mechanistic Insights into Carbon Nanotube Nucleation and Growth on Fe Nanocatalysts in a Thermal Plasma

Hengfei Gu,[a] Stanislav Musikhin,[b] Guangming Cheng,[c] Nan Yao,[c] Yevgeny Raitses,[b] Bruce E. Koel[a, *]

[a]Department of Chemical & Biological Engineering, Princeton University, Princeton, New Jersey 08540, USA
[b]Princeton Plasma Physics Laboratory, Princeton, New Jersey 08543, USA
[c]Princeton Materials Institute, Princeton University, Princeton, New Jersey 08540, USA

*Corresponding author.
Email: bkoel@princeton.edu (Bruce E. Koel)

Materials

Tuball^{TM} graphene nanotubes manufactured by OCSiAl were purchased in powder form (≥80 wt.% SWCNTs as evaluated with an OCSiAl internal method, and ash residue). We used this powder for our measurements and characterization studies reported herein and refer to the as-received powder as soots. N-Methyl-2-pyrrolidinone (1-Methyl-2-pyrrolidinone or NMP) (ACS reagent grade, ≥99.0%, Sigma-Aldrich) was used for debundling of the SWCNTs and separation of the soots. Titanium (Ti) and nickel (Ni) pellets (purity 99.995%, Kurt J. Lesker) were used for thermal evaporation to produce Ti and Ni films. For TEM imaging, PELCO® 300 mesh copper grids were used without any support film and used with single-layer graphene on a Lacey carbon film (Ted Pella, Inc.).

Methods

1. Debundling of soots and transmission electron microscope (TEM) sample preparation

Approximately 0.4 mg of the as-received soots was placed in 20-mL NMP in a Falcon^{TM} tube. The tube was placed in a water bath and ultrasonicated at a power of 480 W and a frequency of 40 kHz for 12 h. Centrifugation at 8000 rpm using a Thermo Scientific Sorvall ST 8 Centrifuge was then carried out to separate the light and heavy components in the NMP after ultrasonication. The resulting NMP liquid that contained visible, loosened soots, was drop casted on TEM grids without any support film. The resulting NMP suspension was drop casted on TEM grids with a monolayer graphene on Lacey carbon support film. In both cases, the TEM grids were dried for 12 h and transferred to a TEM for imaging and analysis.

2. Deposition of Ni and TiN thin films on hydrofluoric acid (HF) etched n$^+$Si wafers as a support for X-ray photoelectron spectroscopy (XPS) analysis

The deposition of TiN on a n$^+$Si substrate was reported in ref. [S1]. It was carried out by thermal evaporation of Ti in an ultrahigh purity N_2 atmosphere. Prior to the thermal evaporation, the Si substrates were cleaned in the following sequence by ultrasonication using acetone, ethanol, and ultrapure Millipore water, for 10 min each, and then were additionally etched by a dilute HF solution with 1:100 volume ratio of HF (48-51%, Fisher Chemical) to Millipore water. The deposition of a 4-nm thick TiN film was carried out by thermal evaporation of Ti in ultrapure N_2 at a pressure of $1.0\text{-}1.2 \times 10^{-4}$ torr. The rate for TiN deposition was 0.8-1.2 Å/s as determined by a quartz crystal microbalance (QCM) using the parameters for thermal evaporation of Ti. Subsequently, thermal evaporation of a 15-nm thick Ni thin film was performed on the 15nmTiN/n$^+$Si substrate in a high vacuum of ~$1.0\text{-}\times 10^{-6}$ torr. The rate for Ni deposition was 0.8-1.2 Å/s as determined by a QCM.

3. Characterization

HRXPS analysis was conducted using a ThermoFisher K-Alpha+ X-Ray Photoelectron Spectrometer set to focus the X-ray beam to a spot size of 400 μm on the as-received soots and the surface of the15nmNi/4nmTiN/n$^+$Si sample. All the spectra are referenced to the adventitious carbon C1s peak at 284.8 eV BE. Raman scattering spectra of the as-received soots on quartz glass were obtained using a Horiba Jobin-Yvon LabRAM Aramis Raman spectrometer with selection of the 532-nm laser. The Titan Cubed Themis S/TEM was used for high-resolution TEM (HRTEM) imaging of carbon nanotubes (CNTs) and Fe nanoparticles (NPs) at an electron acceleration voltage of 300 kV. A Talos F200X S/TEM equipped with a SuperX energy dispersive X-ray spectroscopy (EDXS) system at an electron acceleration voltage of 200 kV was used for high-angle annular dark-field (HAADF) imaging and EDXS mapping and point analysis.

4. Statistical analysis of Fe NP sizes

Bright-field TEM images and the HRTEM images taken on the relatively large, detached Fe NPs and the relatively small, graphene-encapsulated Fe NPs attached on the CNT bundles were analyzed using a method including steps of delineating the 2D shapes of the NPs, extracting the NPs manually from the images, coloring the NPs, and measuring and calculating the equivalent diameters (the diameter of a circle with an area that is equal to that of the NP of interest) of the NPs for statistical analysis. For both cases, the images were obtained on different regions randomly. The number of measured NPs for statistical analysis were 1875 for detached Fe NPs and 202 for Fe NPs attached on the CNT bundles.

5. 2D curvature measurements

The 2D curvatures of Fe NP surfaces were measured from the HRTEM images using the open-source Fiji plugin "Kappa" that uses a procedure on the basis of cubic B-splines.[1]

6. Molecular dynamics (MD) simulations

CNT models were created using the program Nanotube Modeler.[2] The simulation box was 200 Å in x, y, and z directions. The LAMMPS code was used for MD simulations.[3] Periodic boundary conditions in three dimensions were applied. The adaptive intermolecular reactive empirical bond order (AIREBO) potential was applied in these simulations.[4] The MD timestep used was 1 fs. The simulation box with a CNT of interest was equilibrated at 300 K, heated up to a target temperature, and maintained at the target temperature for 10 ps. The NPT ensemble (with constant particle number, pressure, and temperature) was applied. No external pressure was applied.

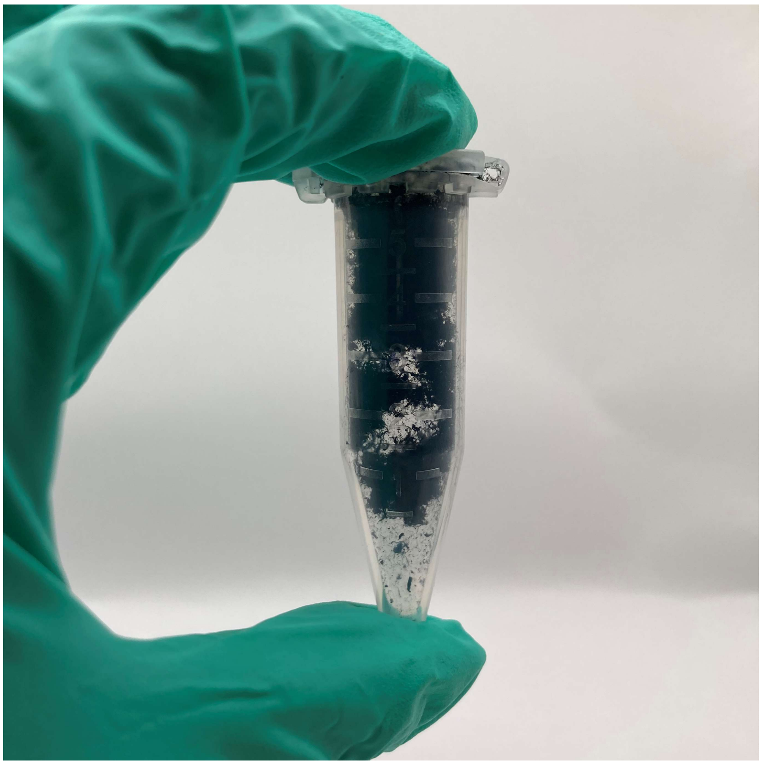

Figure S1. Photograph of as-received soots for Tuball^{TM} graphene nanotubes manufactured by OCSiAl.

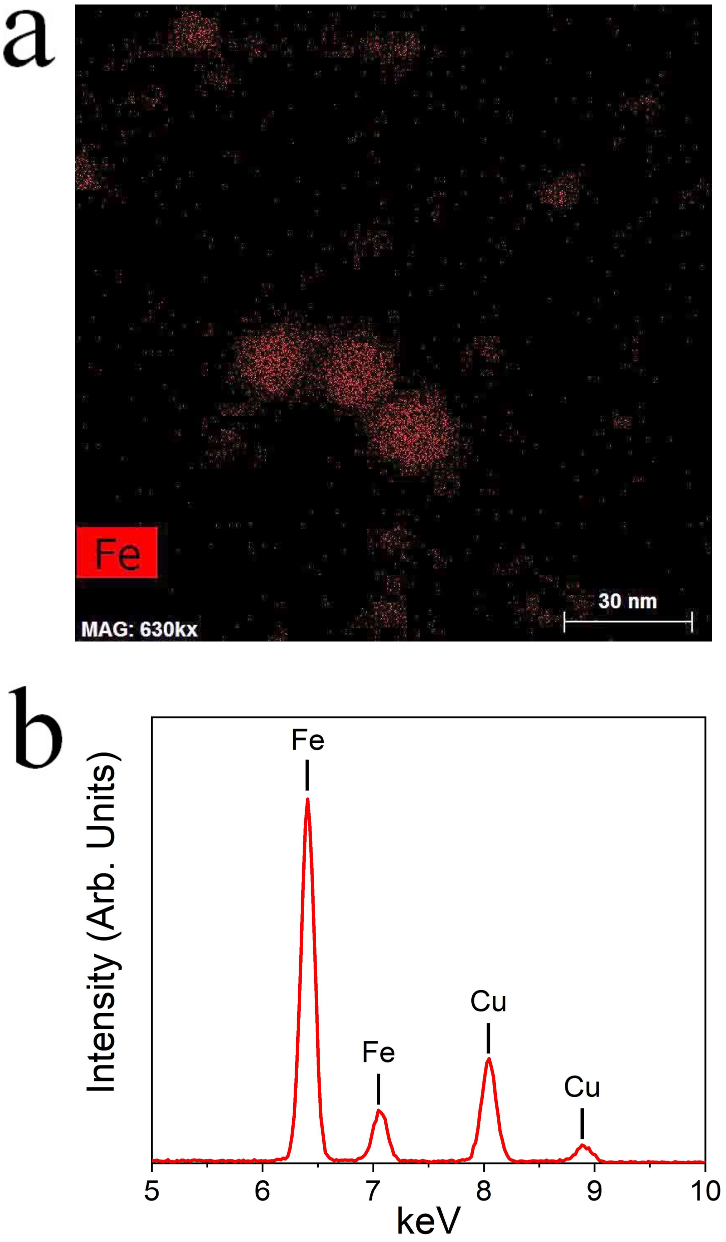

Figure S2. **(a)** EDXS Fe mapping corresponding to **Figure 1c** in the main text. **(b)** EDXS point analysis of the bright spot indicated by the yellow arrow in **Figure 1c**, showing Fe peaks, as well as Cu peaks that arise from the Cu TEM grid.

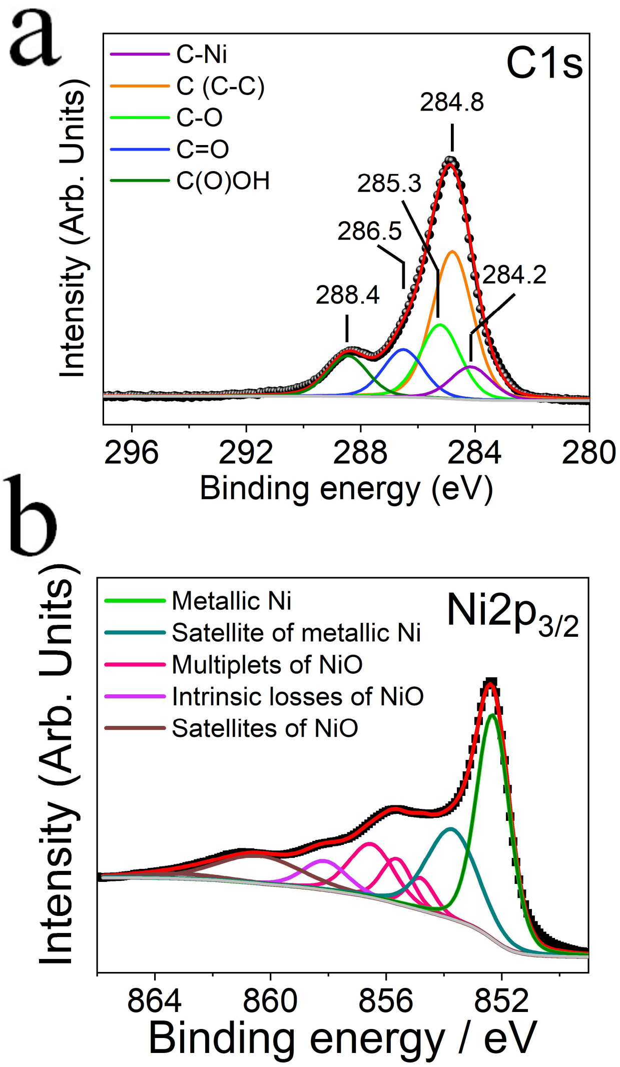

Figure S3. (a) C1s and (b) Fe2$p_{3/2}$ XPS spectra of the 15nmNi/4nmTiN/n$^+$Si substrate that was used as a support for the soots.

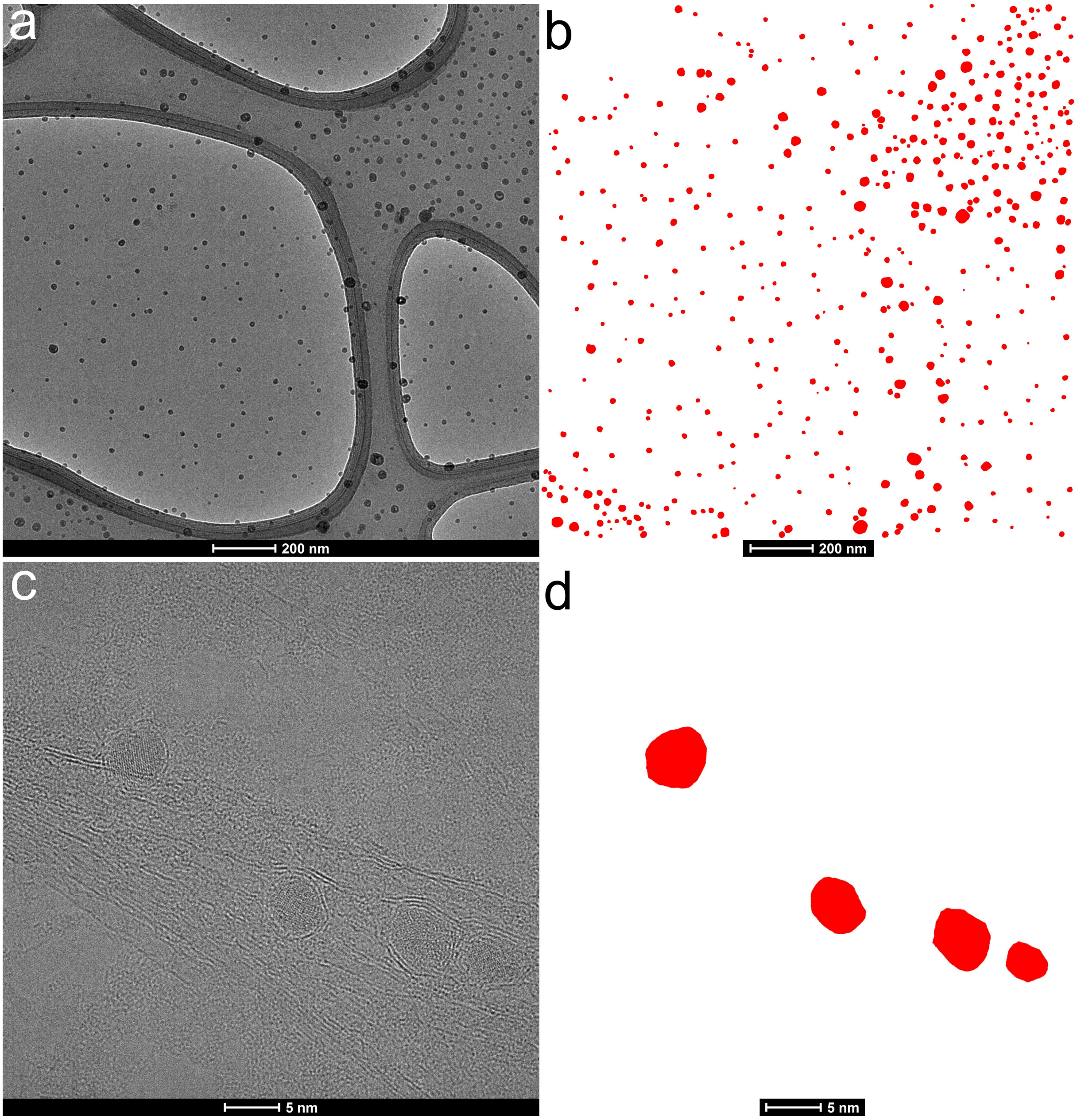

Figure S4. **(a)** A representative bright field TEM image of detached Fe NPs and **(c)** a representative HRTEM image of graphene-encapsulated Fe NPs attached to a CNT bundle, which were used for statistical analysis of Fe NP sizes. **(b)** and **(d)** show images of manually extracted Fe NPs corresponding to the images shown in **(a)** and **(c),** respectively.

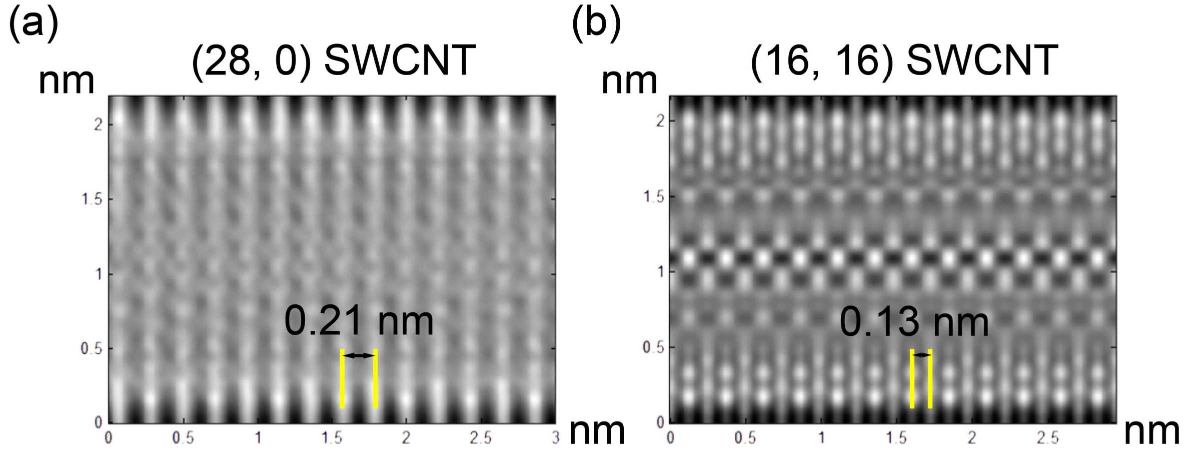

Figure S5. TEM images of **(a)** (28, 0) zigzag SWCNT and **(b)** (16, 16) armchair SWCNT with a diameter of near 2.2 nm and a length of near 3 nm simulated under a 300 keV electron beam using the QSTEM code. The horizontal axis is along the long axis of the SWCNT, while the vertical axis is parallel to the cross section of the SWCNT.

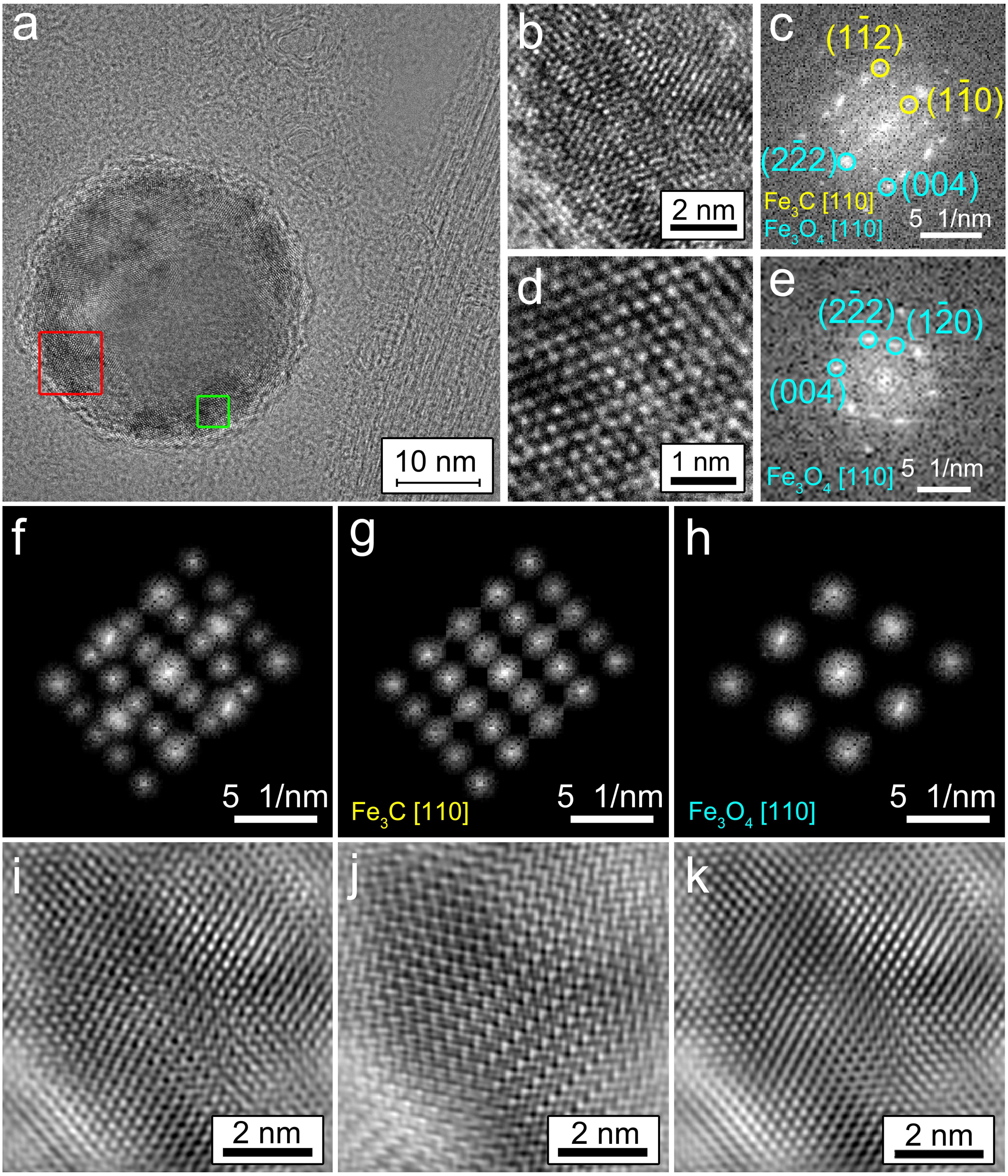

Figure S6. **(a)** HRTEM image of a detached Fe NP near a CNT bundle. **(b)** and **(d)** Zoomed-in HRTEM images of the regions in **(a)** indicated by the red and green boxes, respectively. **(c)** and **(e)** display FFT patterns of the whole images displayed in **(b)** and **(d)**, respectively. **(f)** Masked FFT pattern of the Fourier transform spots shown in **(c)**; **(g)** and **(h)** Masked FFT patterns of the Fourier transform spots for Fe_3C and Fe_3O_4 shown in **(c)**, respectively. **(i)**-**(k)** IFFT images corresponding to the masked FFT patterns shown in **(f)**-**(h)**, respectively.

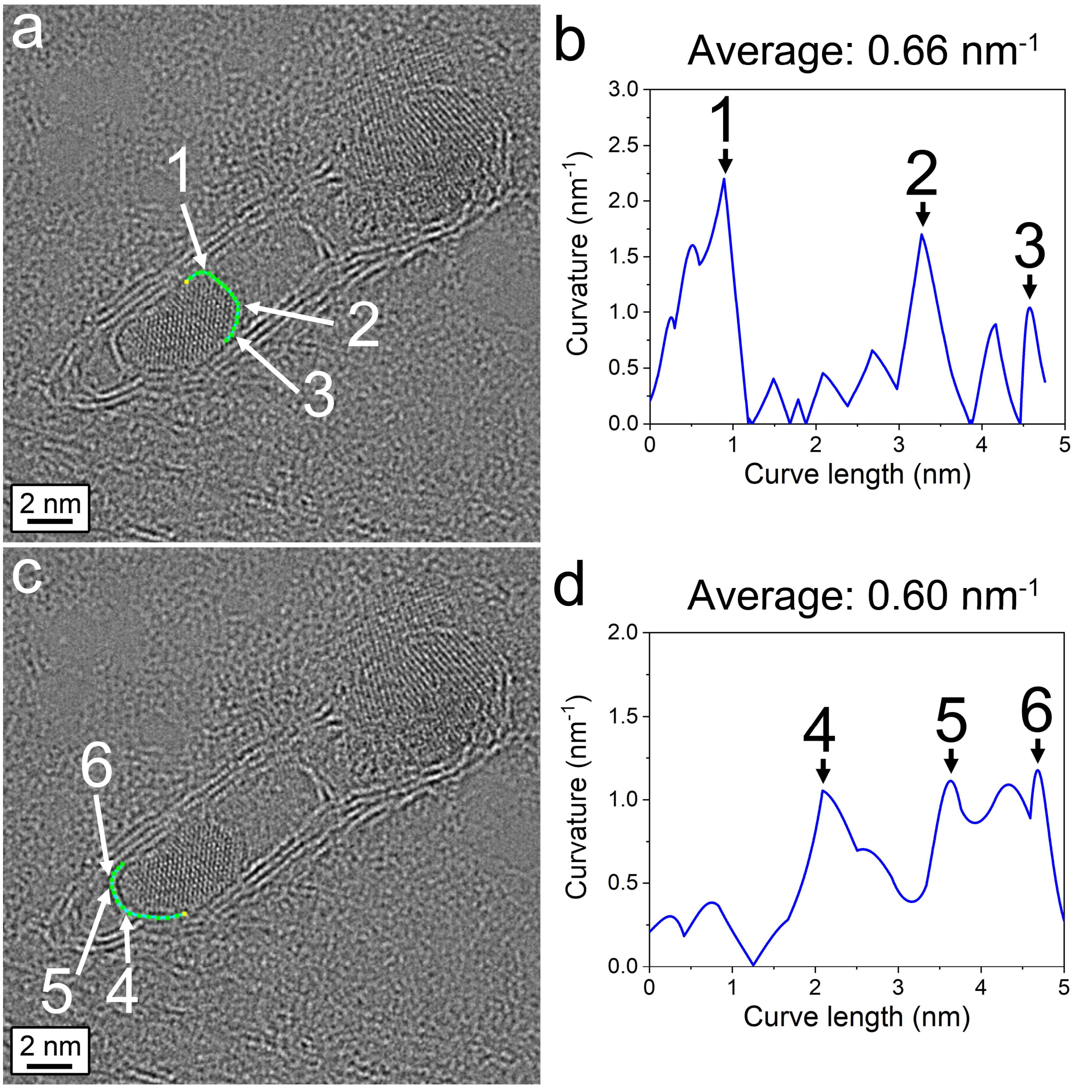

Figure S7. (a) and **(c)** NP surfaces used for curvature measurement are indicated by curves denoted by green squares (the yellow square shows the starting measurement point) on the HRTEM image shown in **Figure 5a** in the main text. **(b)** and **(d)** Curvature values along the curves highlighted in the HRTEM images shown in **(a)** and **(c)**, respectively.

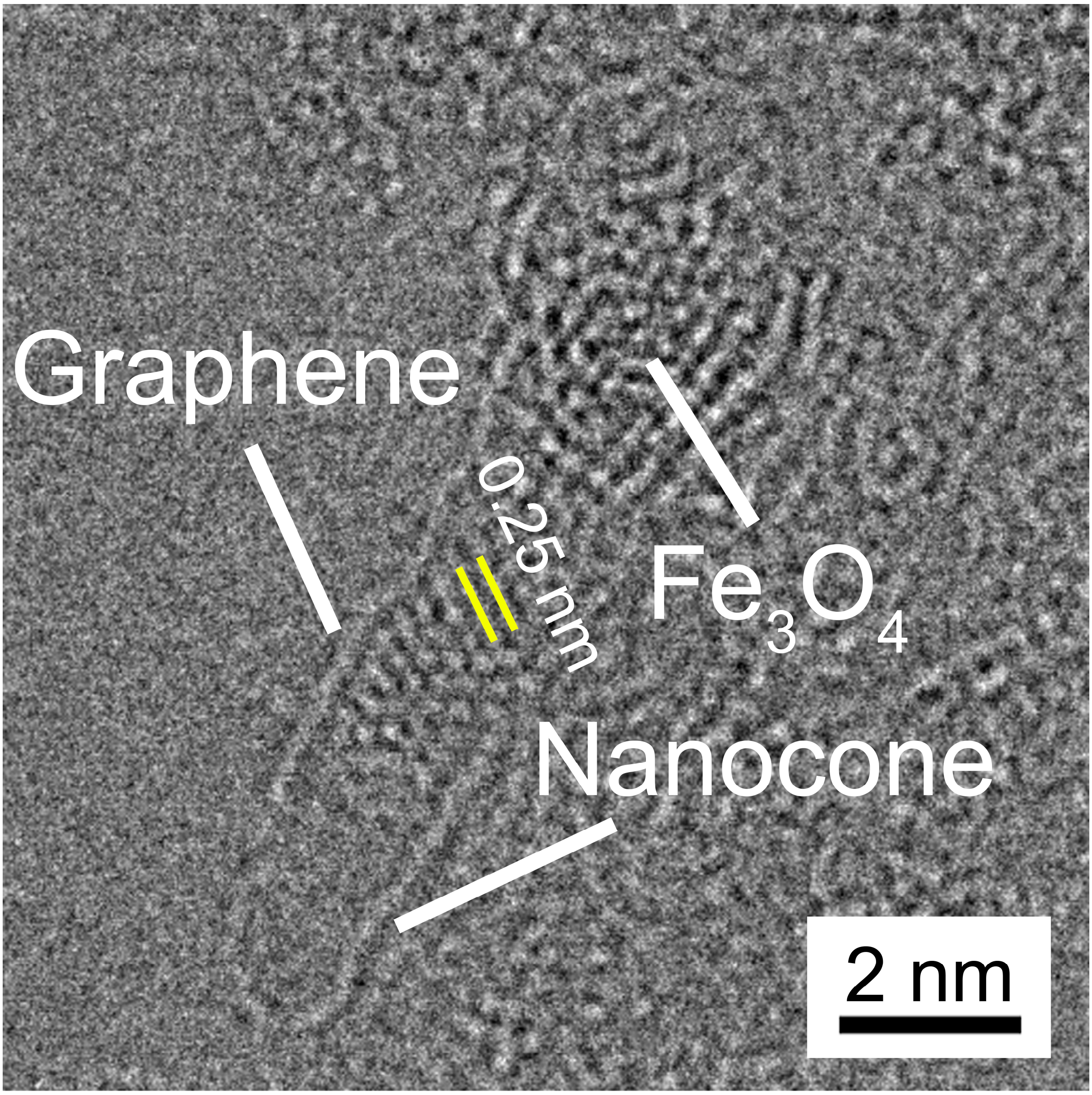

Figure S8. HRTEM image of a SWCNC grown on the narrower end of an ultrasmall molten Fe nanodroplet that was oxidized from its wider end to Fe_3O_4.

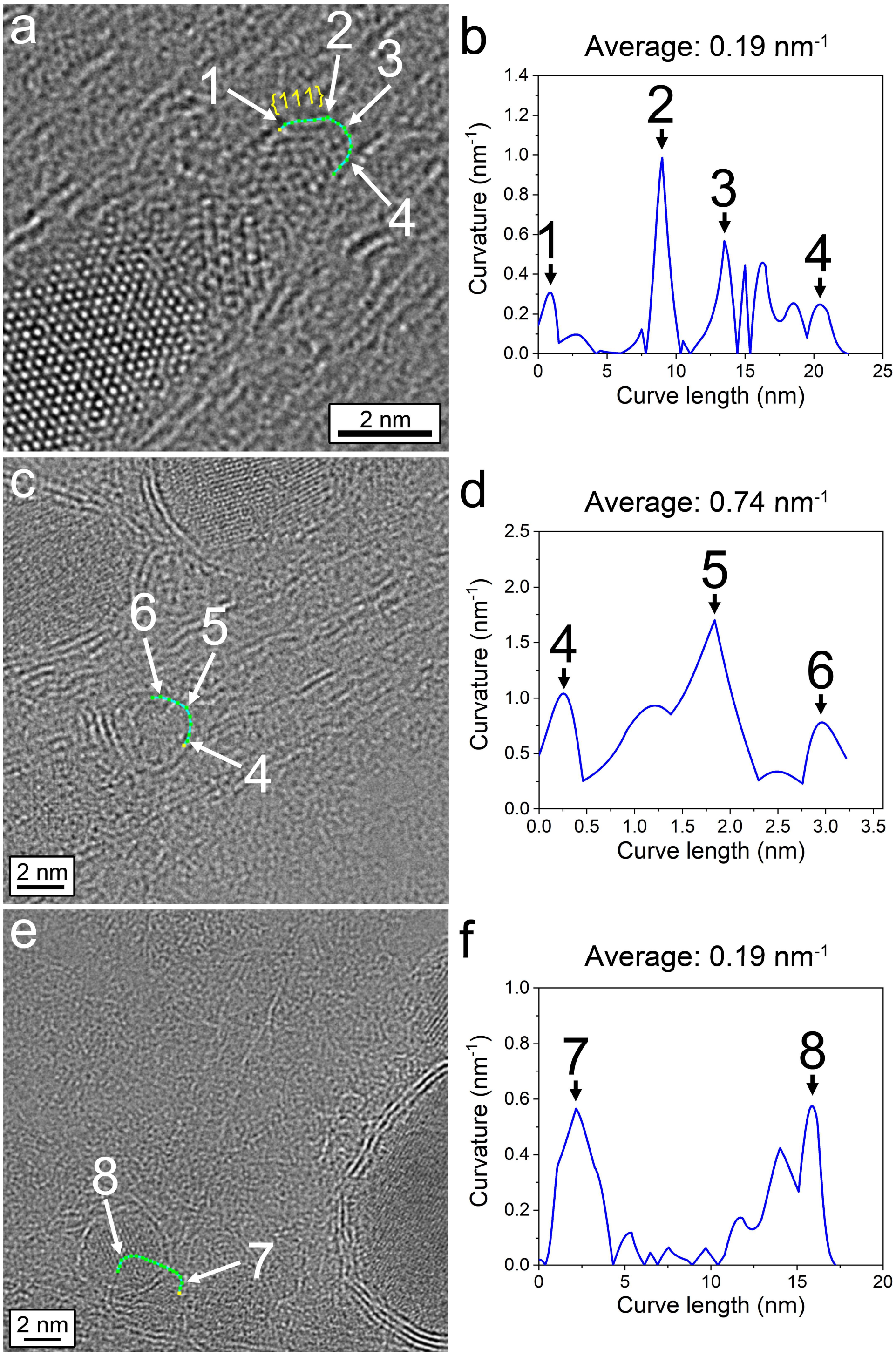

Figure S9. (a), **(c),** and **(e)** NP surfaces used for curvature measurement denoted by green squares (the yellow square shows the starting measurement point) on the HRTEM images shown in **Figures 6b, 6e, and 6g** in the main text, respectively. **(b)**, **(d)** and **(f)** Curvature values along the curves highlighted in the HRTEM images shown in **(a)**, **(c),** and **(e)**, respectively.

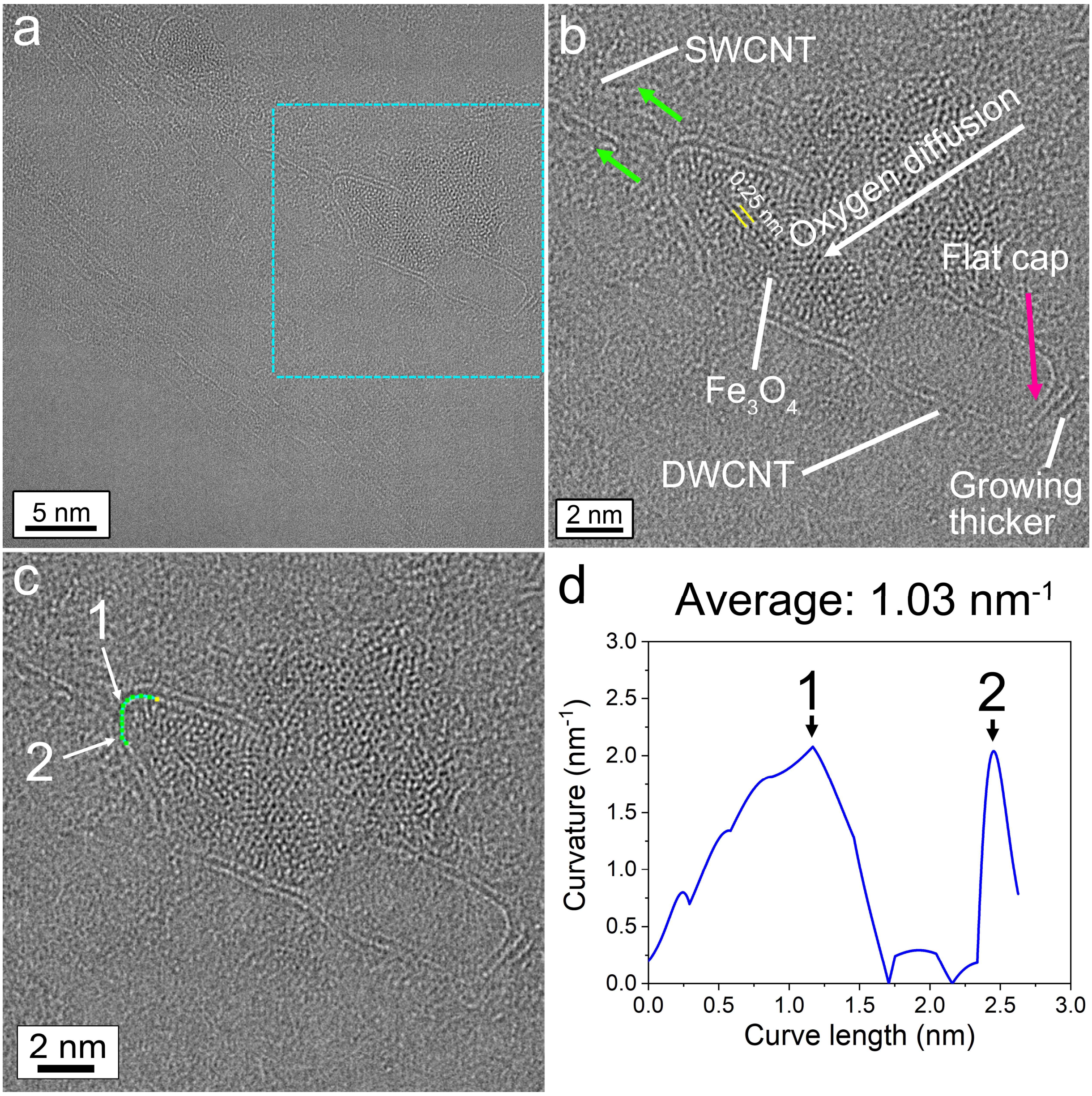

Figure S10. (a) HRTEM image of a Fe NP that was fully oxidized to Fe_3O_4 from its body side with a SWCNT growing out from the second layer of graphene on its sharp end, and a DWCNT growing out from its wider end that has a flat graphene termination. **(b)** Zoomed-in HRTEM image of the region defined by the turquoise box shown in **(a). (c)** NP surface used for curvature measurement denoted by green squares (the yellow square shows the starting measurement point) on the HRTEM image shown in **(b)**. **(d)** Curvature values along the curve highlighted in the HRTEM image shown in **(c)**.

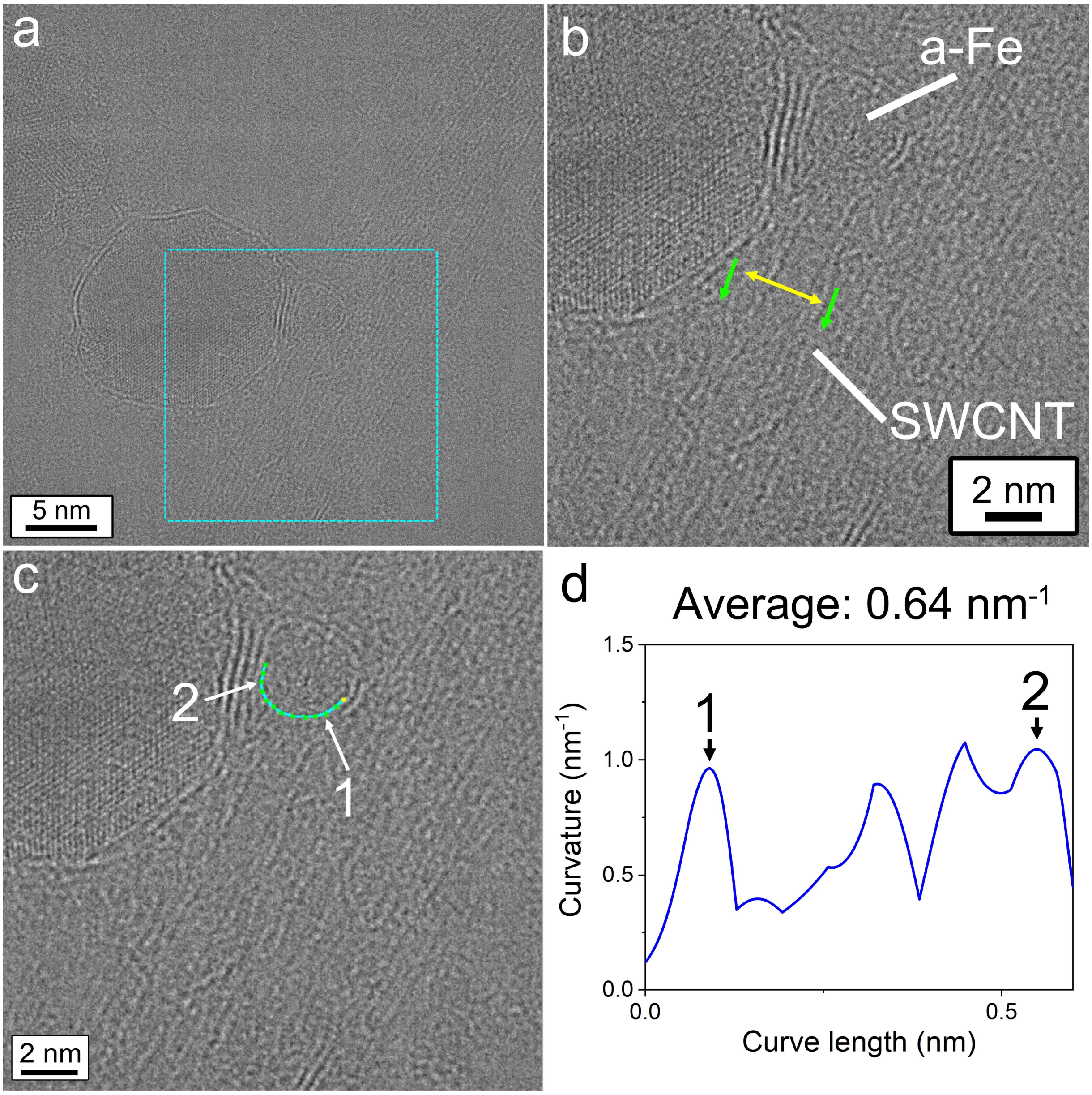

Figure S11. (a) HRTEM image of an ultrasmall, graphene-encapsulated, amorphous Fe NP with a SWCNT growing out from its narrower end. **(b)** Zoomed-in HRTEM image of the region defined by the turquoise box shown in **(a). (c)** NP surface used for curvature measurement denoted by green squares (the yellow square shows the starting measurement point) on the HRTEM image shown in **(b)**. **(d)** Curvature values along the curve highlighted in the HRTEM image shown in **(c)**.

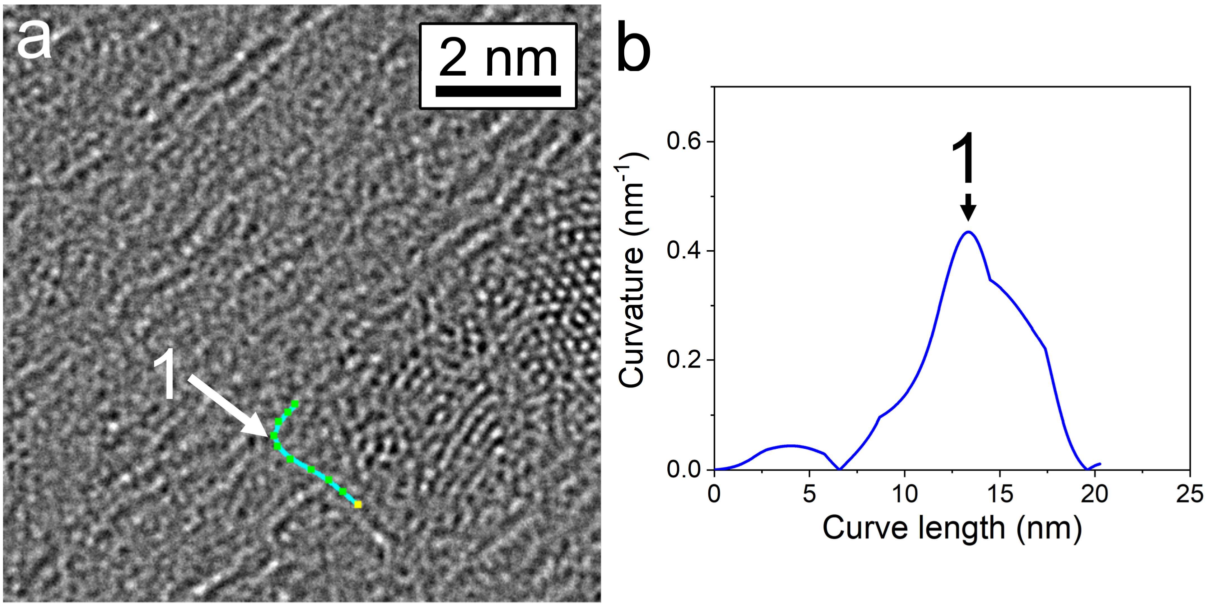

Figure S12. (a) NP surface used for curvature measurement denoted by green squares (the yellow square shows the starting measurement point) on the HRTEM image of the wider end of the Fe NP shown in **Figure 6a** in the main text. **(b)** Curvature values along the curve highlighted in the HRTEM images shown in **(a)**.

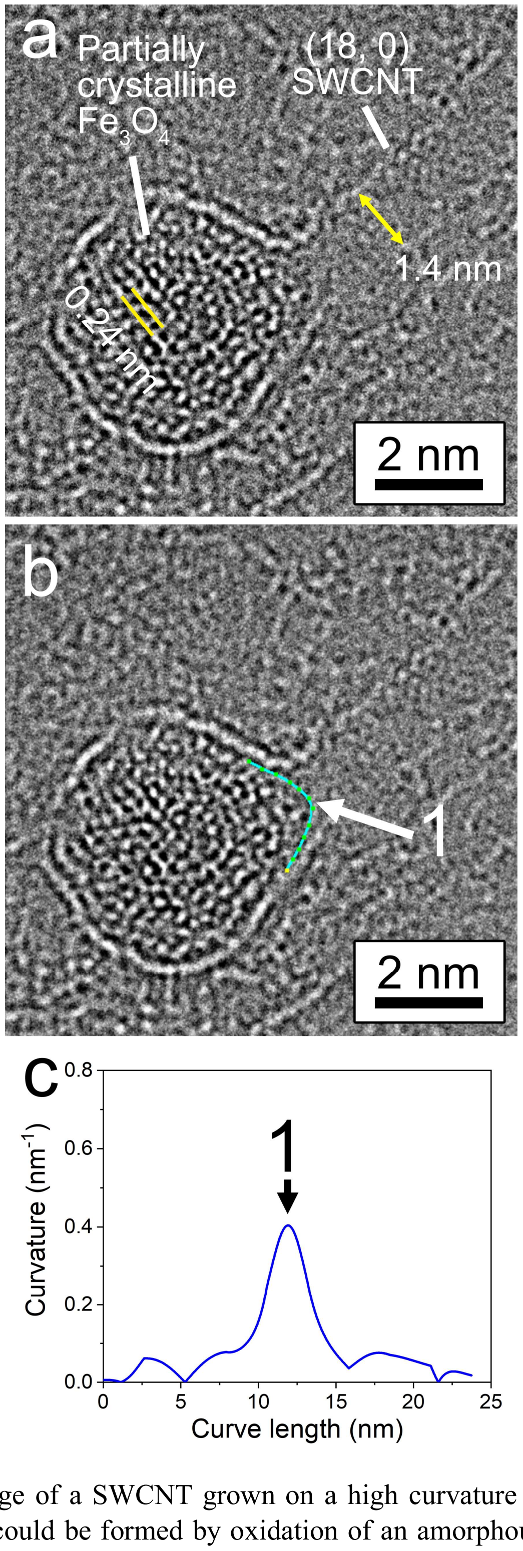

Figure S13. (a) HRTEM image of a SWCNT grown on a high curvature corner of a pumpkin-shaped, amorphous Fe_3O_4 NP, which could be formed by oxidation of an amorphous Fe NP. **(b)** The NP surface used for curvature measurement is denoted by green squares (the yellow square shows the starting measurement point) on the HRTEM image shown in **(b)**. **(c)** Curvature values along the curve highlighted in the HRTEM images shown in **(b)**.

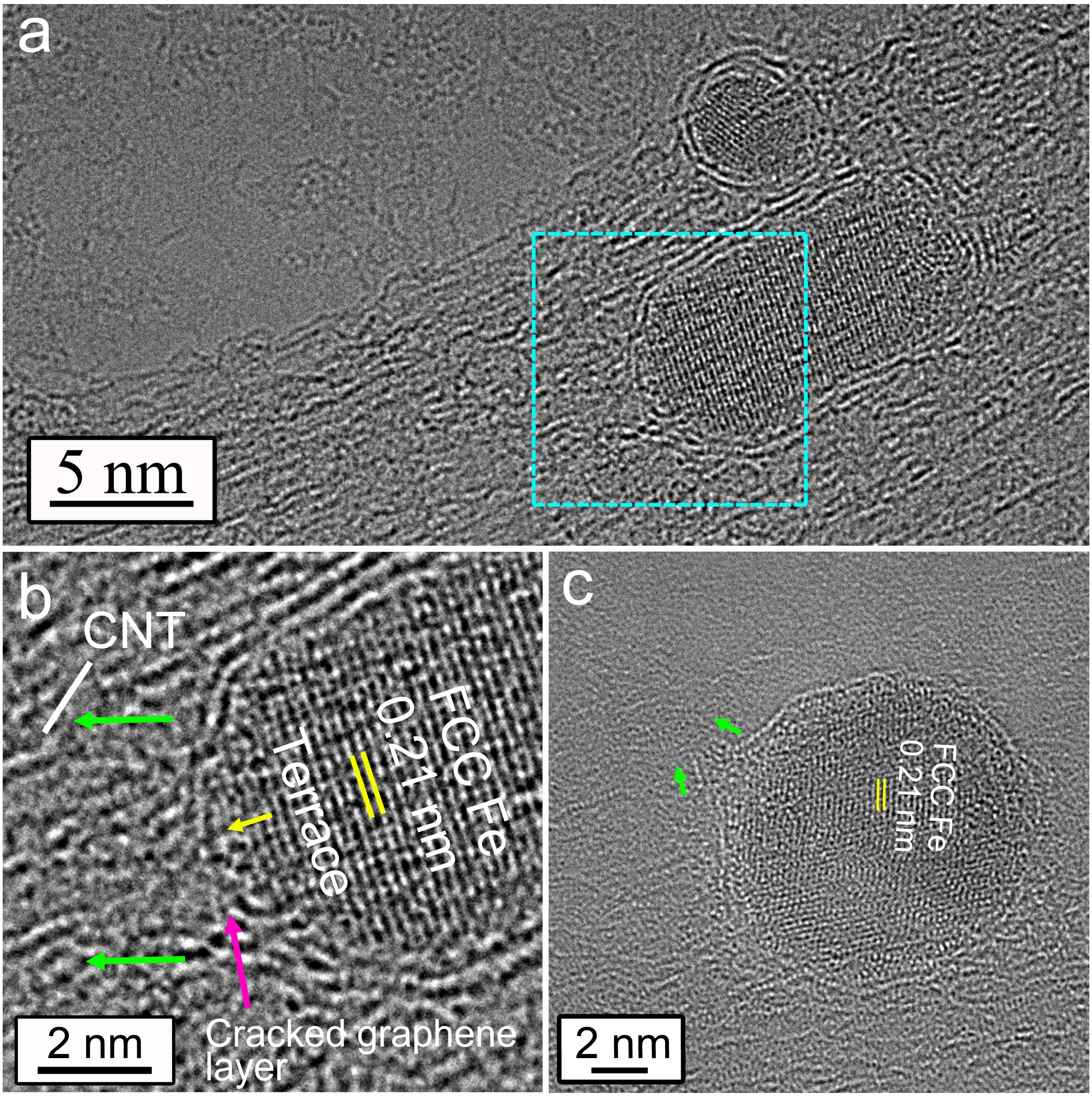

Figure S14. (a) HRTEM image of a graphene-encapsulated, bar-shaped FCC Fe NP attached to a CNT bundle. **(b)** Zoomed-in HRTEM image of the left end of the Fe NP in the region defined by the turquoise box shown in **(a)**. One explanation for this structure is that a FCC (111) Fe terrace formed during crystallization of molten Fe to FCC Fe within the graphene encapsulation, causing cracking of the graphene covering and growth of a tubular structure from the second or outer graphene encapsulating layer. **(c)** HRTEM image of a FCC Fe NP encapsulated by one layer of graphene. A similar example is shown in **(c)**. The preferred growth of the FCC {111} planes during crystallization of molten Fe caused the cracking of the graphene encapsulation layer at the sharp facet kink or step. An open tubular structure started to grow out from the opening of the graphene covering. Meanwhile, O diffused into the tubular structure to oxidize Fe.

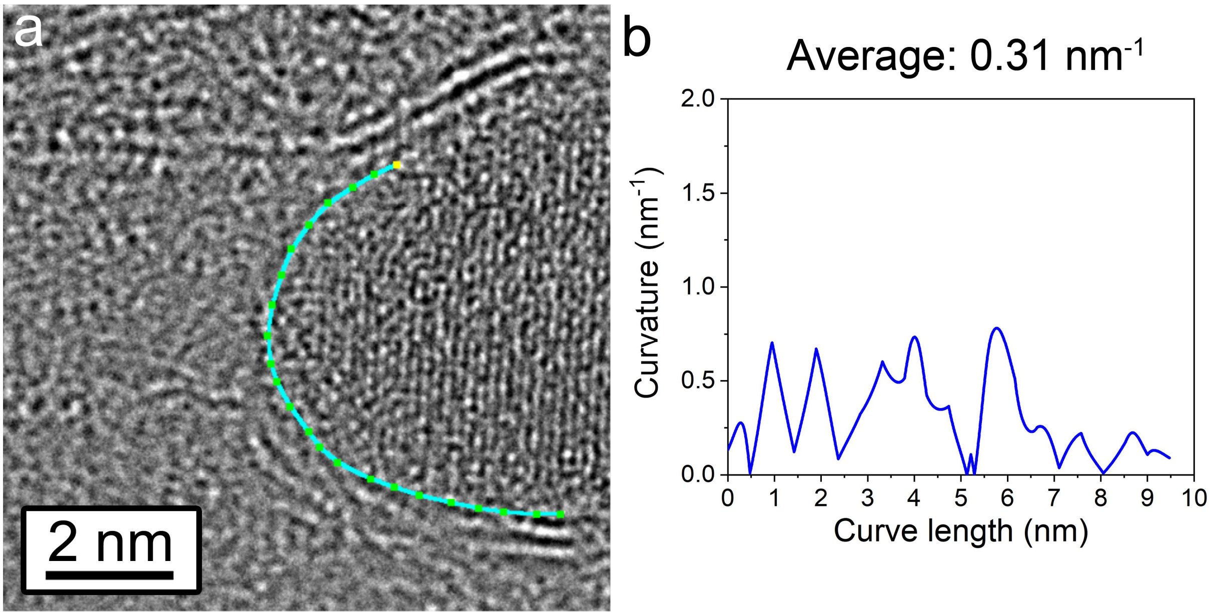

Figure S15. (a) The NP surface used for curvature measurement is denoted by the green squares (the yellow square is the starting measurement point) on the HRTEM image shown in **Figure 7e** in the main text. **(b)** Curvature values along the curve highlighted in the HRTEM image shown in **(a)**.

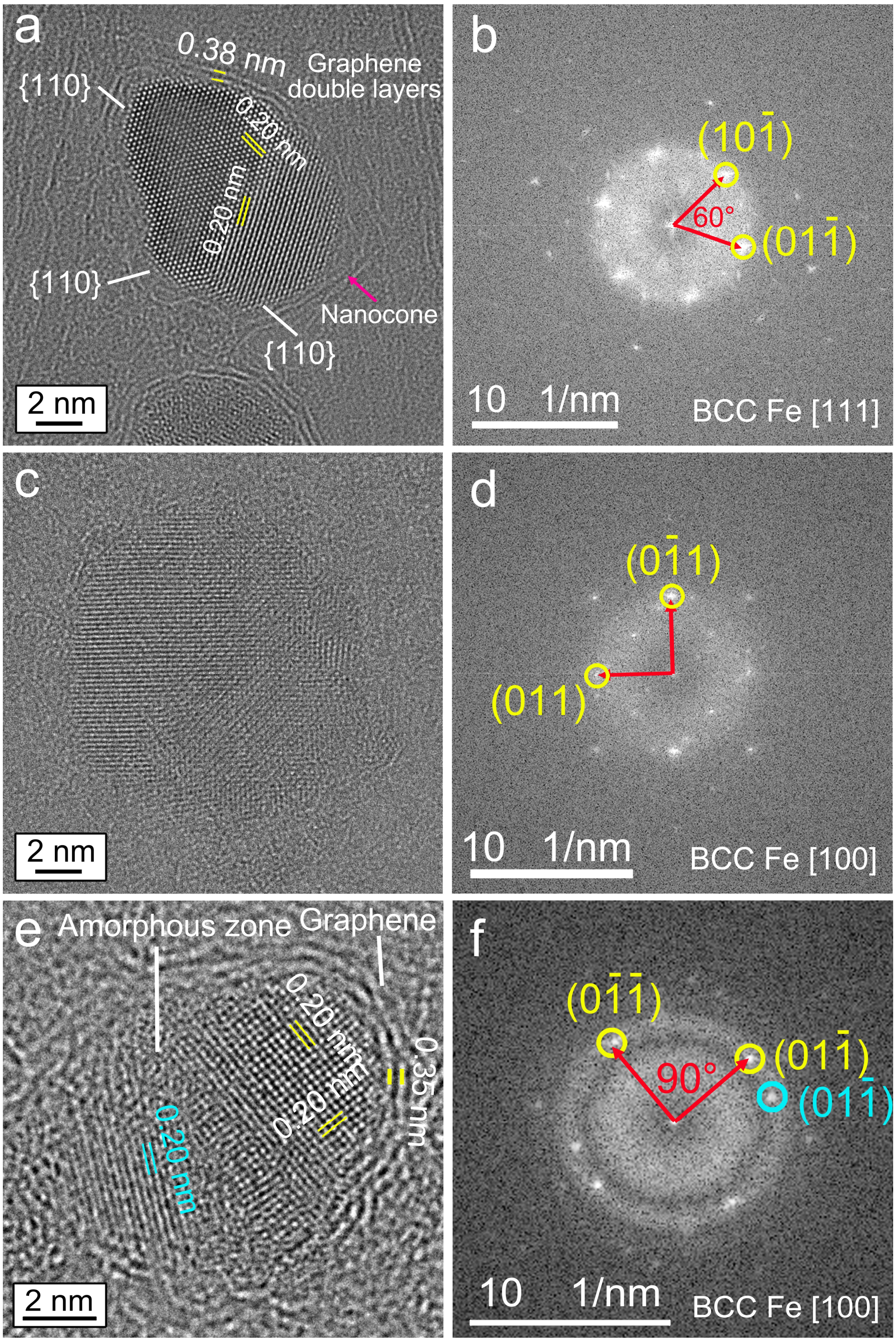

Figure S16. Images and analysis used to identify BCC Fe NPs. **(a)**, **(c)** and **(e)** show HRTEM images of BCC Fe NPs with and without graphene encapsulation. **(b)**, **(d),** and **(f)** display FFT patterns of the whole images displayed in **(a)**, **(c)** and **(e)**, respectively.

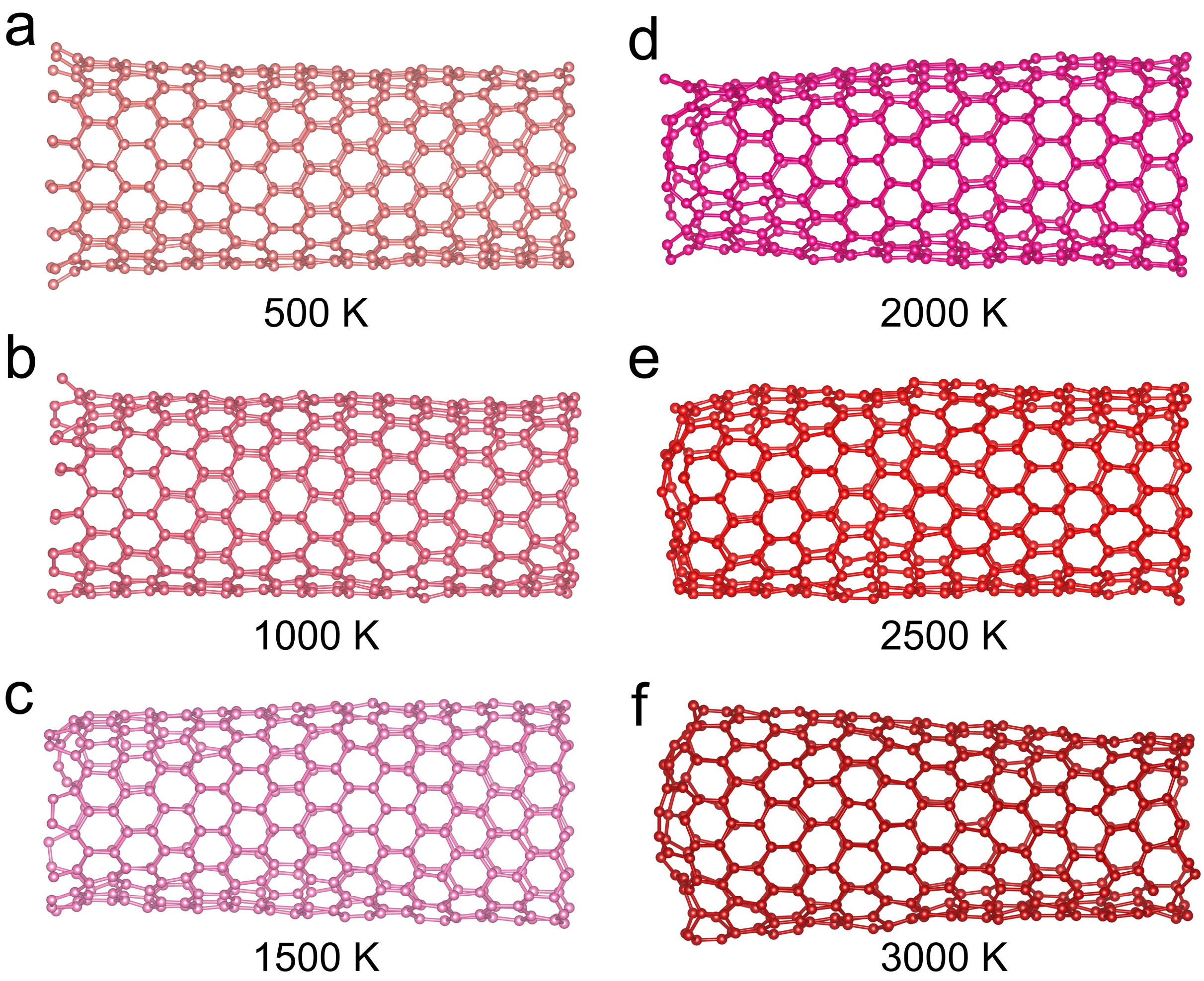

Figure S17. Side views of a (14, 0) SWCNT that is 3 nm in length after MD simulations of annealing in vacuum at 500 K to 3000 K. At 500 K, the left-hand side of the tube has a C-zigzag termination and the right-hand side of the tube has a zigzag termination.

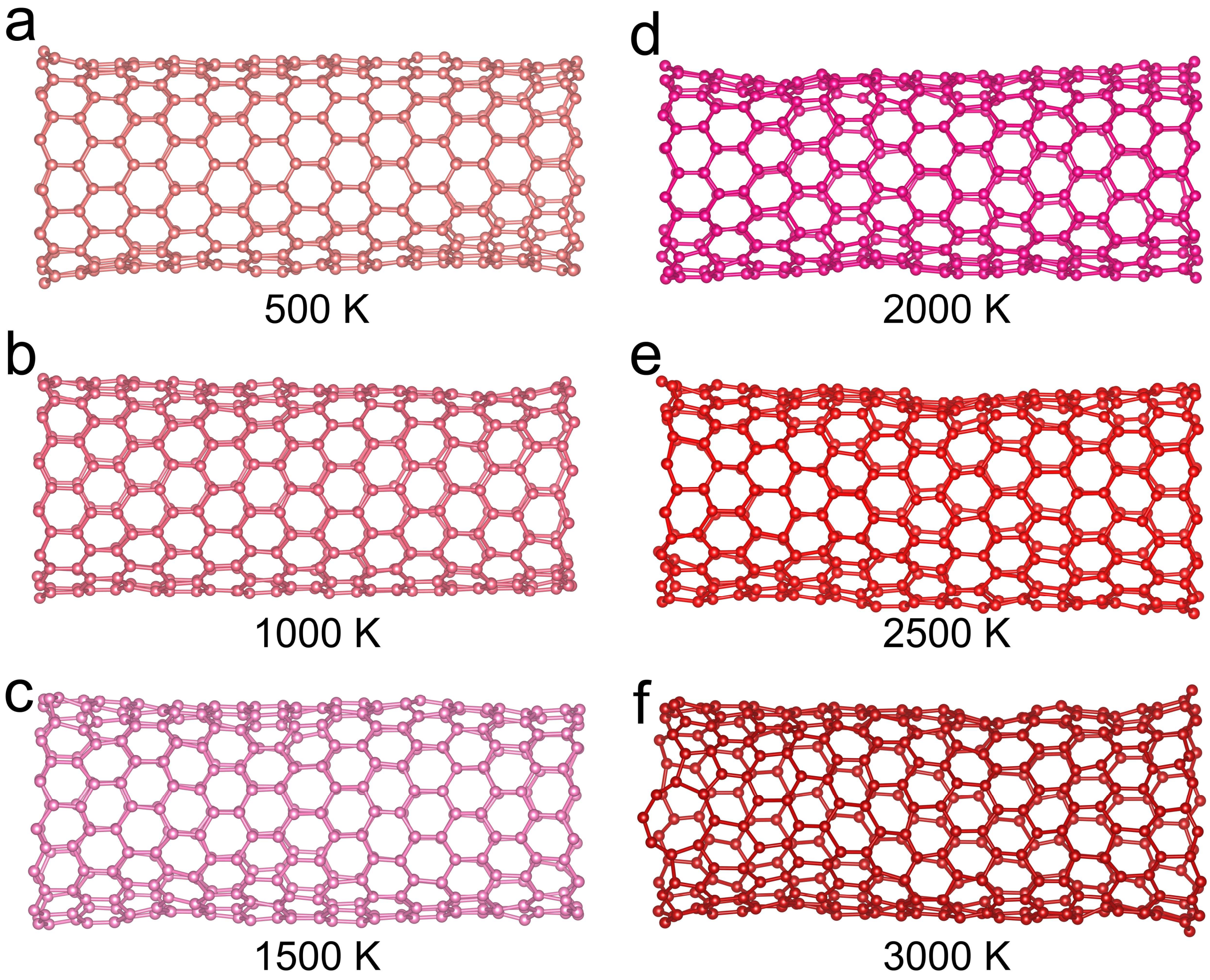

Figure S18. Side views of a (14, 0) SWCNT 3 nm in length with both zigzag terminations after MD simulated annealing in vacuum at a temperature from 500 K to 3000 K. No capping was observed.

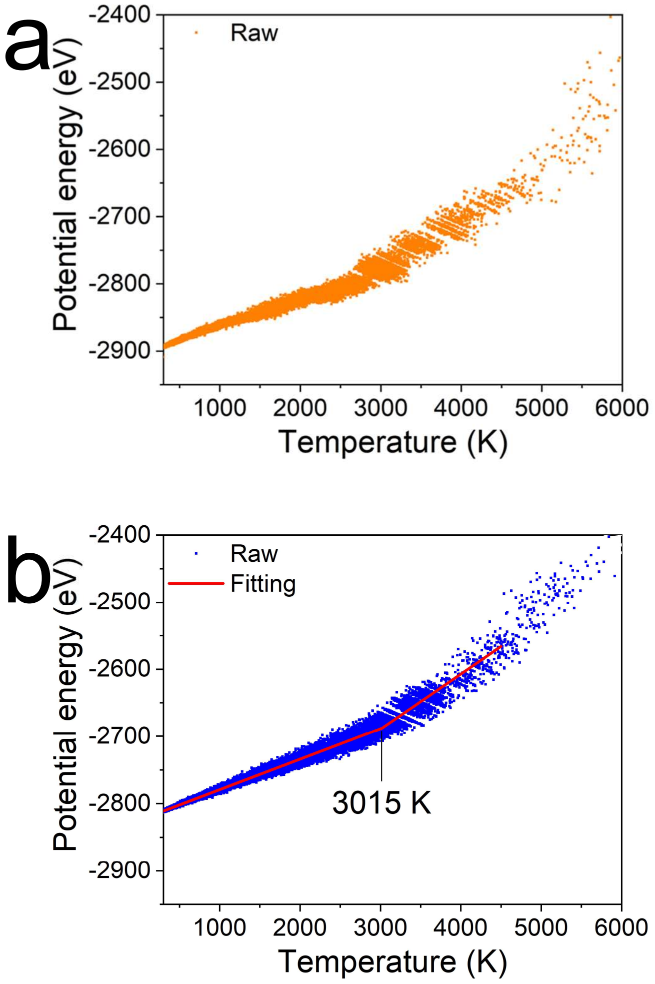

Figure S19. Potential energy (PE) as a function of temperature, PE(T) obtained during MD simulated heating of a (14, 0) SWCNT 3 nm in length with (a) a zigzag termination on one end and a C-zigzag termination on the other end, and (b) zigzag terminations on both ends. Note that the slope of PE(T) indicates the heat capacity.

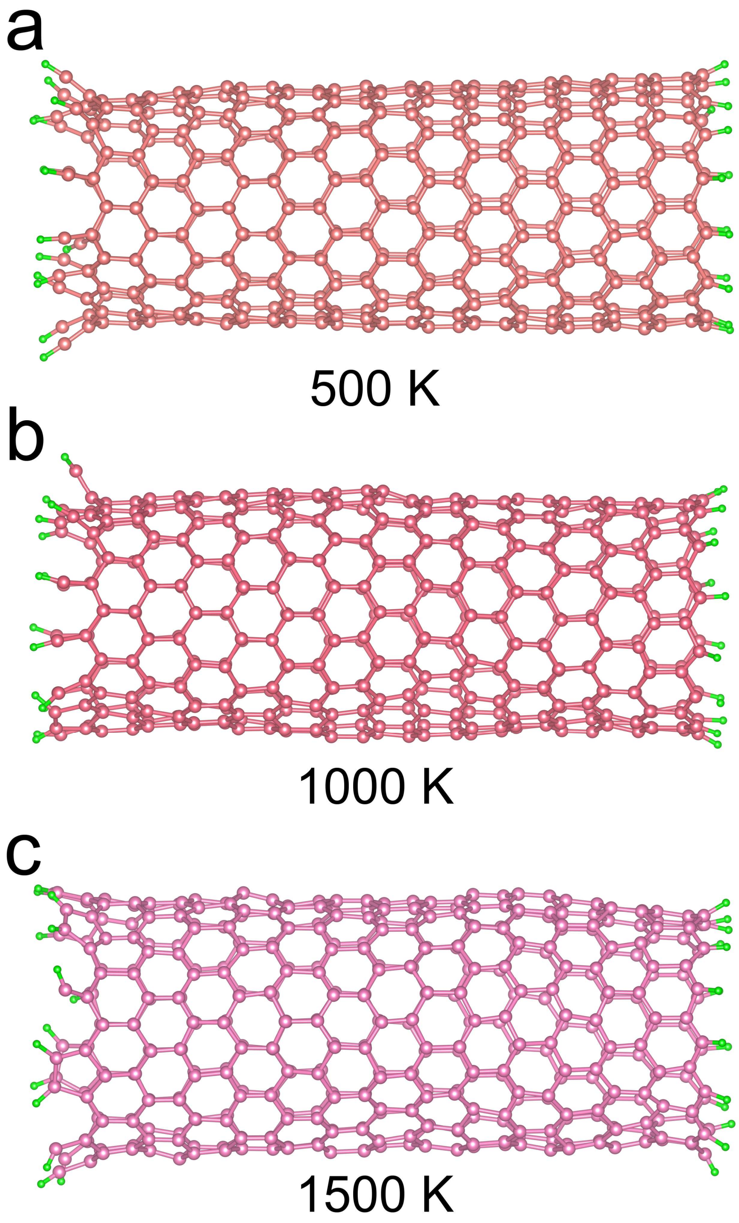

Figure S20. Side views of a (14, 0) SWCNT that is 3 nm in length after MD simulations of annealing in vacuum at 500 K to 1500 K. At 500 K, the left-hand side of the tube has a CH-zigzag termination and the right-hand side of the tube has a H-zigzag termination.

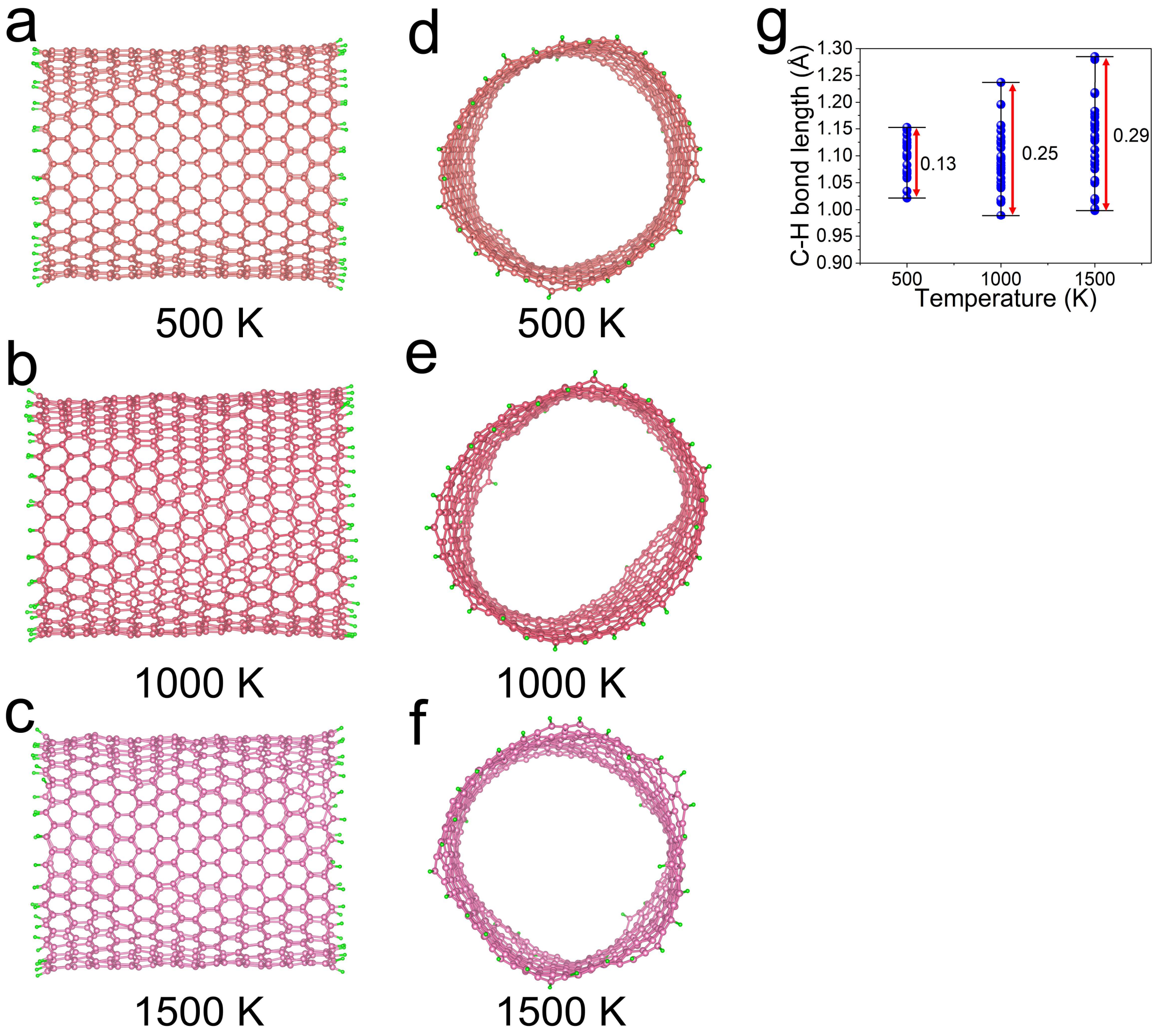

Figure S21. **(a)-(c)** Side views of a (28, 0) SWCNT 3 nm in length after MD simulations of annealing in vacuum at 500 K to 1500 K. At 500 K, both ends of the tube had H-zigzag terminations. **(d)-(f)** End-on views of the same tubes as shown in **(a)-(c)**. **(g)** Instantaneous values of all the C-H bond lengths at the end of the MD simulation for the H-zigzag termination of the (28,0) SWCNT as a function of temperature from 500 K to 1500 K.

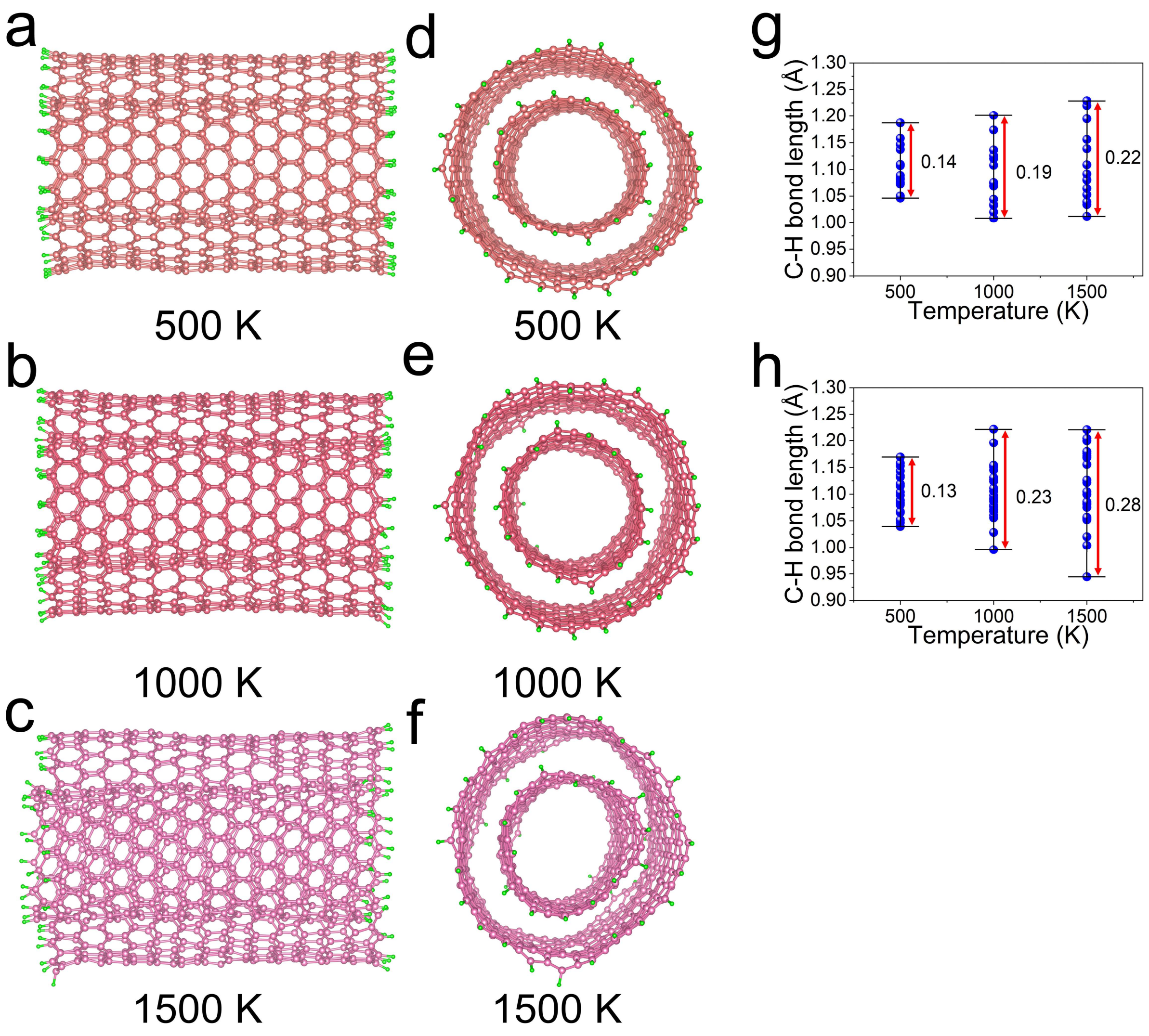

Figure S22. (a)-(c) Side views of a (24, 0)/(14, 0) DWCNT that is 3 nm in length after MD simulations of annealing in vacuum at 500 K to 1500 K. At 500 K, both ends of the tube had H-zigzag terminations. **(d)-(f)** End-on views of the same tubes as shown in **(a)-(c)**. Instantaneous values of all the C-H bond lengths at the end of the MD simulation for the H-zigzag terminations of **(g)** the inner (14, 0) CNT and **(h)** the outer (24, 0) CNT as a function of temperature from 500 K to 1500 K.

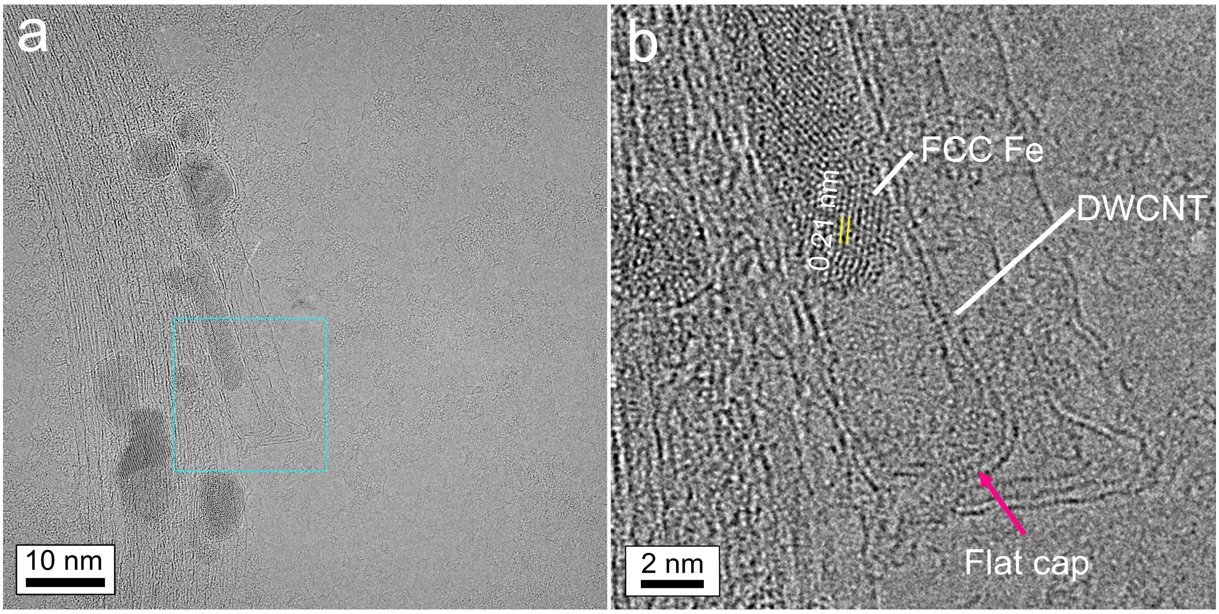

Figure S23. (a) HRTEM image of a bar-shaped FCC Fe NP with a DWCNT that has grown out from its cylindrical body that was terminated by a flat graphene layer. **(b)** Zoomed-in HRTEM image of the region defined by the turquoise box shown in **(a)**.

References

(S1) Brouhard, H. M. G. J. Kappa (κ): Analysis of Curvature in Biological Image Data using B-splines. *bioRxiv* **2019**, 852772. DOI: https:// doi. org/ 10. 1101/ 852772.

(S2) Melchor, S.; Dobado, J. A. CoNTub: An algorithm for connecting two arbitrary carbon nanotubes. *Journal of Chemical Information and Computer Sciences* **2004**, *44* (5), 1639-1646.

(S3) Thompson, A. P.; Aktulga, H. M.; Berger, R.; Bolintineanu, D. S.; Brown, W. M.; Crozier, P. S.; In't Veld, P. J.; Kohlmeyer, A.; Moore, S. G.; Nguyen, T. D. LAMMPS-a flexible simulation tool for particle-based materials modeling at the atomic, meso, and continuum scales. *Computer Physics Communications* **2022**, *271*, 108171.

(S4) Stuart, S. J.; Tutein, A. B.; Harrison, J. A. A reactive potential for hydrocarbons with intermolecular interactions. *The Journal of Chemical Physics* **2000**, *112* (14), 6472-6486.
